# Recent advancements in the breeding of sorghum crop: current status and future strategies for marker-assisted breeding

**DOI:** 10.3389/fgene.2023.1150616

**Published:** 2023-05-11

**Authors:** Faheem Shehzad Baloch, Muhammad Tanveer Altaf, Waqas Liaqat, Mehmet Bedir, Muhammad Azhar Nadeem, Gönül Cömertpay, Nergiz Çoban, Ephrem Habyarimana, Celaleddin Barutçular, Ibrahim Cerit, Ndomelele Ludidi, Tolga Karaköy, Muhammad Aasim, Yong Suk Chung, Muhammad Amjad Nawaz, Rüştü Hatipoğlu, Kağan Kökten, Hyeon-Jin Sun

**Affiliations:** ^1^ Faculty of Agricultural Sciences and Technologies, Sivas University of Science and Technology, Sivas, Türkiye; ^2^ Department of Field Crops, Faculty of Agriculture, Çukurova University, Adana, Türkiye; ^3^ Eastern Mediterranean Agricultural Research Institute, Adana, Türkiye; ^4^ International Crops Research Institute for the Semi-Arid Tropics, Hyderabad, Telangana, India; ^5^ Plant Stress Tolerance Laboratory, Department of Biotechnology, University of the Western Cape, Bellville, South Africa; ^6^ DSI-NRF Centre of Excellence in Food Security, University of the Western Cape, Bellville, South Africa; ^7^ Department of Plant Resources and Environment, Jeju National University, Jeju, Republic of Korea; ^8^ Advanced Engineering School (Agrobiotek), Tomsk State University, Tomsk, Russia; ^9^ Kırşehir Ahi Evran Universitesi Ziraat Fakultesi Tarla Bitkileri Bolumu, Kırşehir, Türkiye; ^10^ Subtropical Horticulture Research Institute, Jeju National University, Jeju, Republic of Korea

**Keywords:** Sorghum, marker-assisted breeding, genomics, genomics prediction, molecular breeding

## Abstract

Sorghum is emerging as a model crop for functional genetics and genomics of tropical grasses with abundant uses, including food, feed, and fuel, among others. It is currently the fifth most significant primary cereal crop. Crops are subjected to various biotic and abiotic stresses, which negatively impact on agricultural production. Developing high-yielding, disease-resistant, and climate-resilient cultivars can be achieved through marker-assisted breeding. Such selection has considerably reduced the time to market new crop varieties adapted to challenging conditions. In the recent years, extensive knowledge was gained about genetic markers. We are providing an overview of current advances in sorghum breeding initiatives, with a special focus on early breeders who may not be familiar with DNA markers. Advancements in molecular plant breeding, genetics, genomics selection, and genome editing have contributed to a thorough understanding of DNA markers, provided various proofs of the genetic variety accessible in crop plants, and have substantially enhanced plant breeding technologies. Marker-assisted selection has accelerated and precised the plant breeding process, empowering plant breeders all around the world.

## Introduction

Sorghum (*Sorghum bicolor* L.) is one annual grass from the Poaceae family with C4 metabolism. The plant first appeared 8,000–5,000 B.C. in Northeastern Africa (Mann et al., 1983). That region contains the greatest diversity of cultivated and wild sorghum species. Posterior cultivation was found 4,500 B.C. in Western Rojdi, Saurashtra, India (Vavilov et al., 1992; Damania, 2002). That cereal is the fifth most important cereal crop in the world. Sorghum is a rich protein and fiber source (Khalid et al., 2022), and it has great calcium, phosphorus and potassium, but low sodium contents compared to rice and wheat (Rao, 2019). Preparations based on sorghum flour (i.e., bread, biscuit, pasta, pastry, and porridge) are suitable for a gluten-free diet. Sorghum is not only a major food crop, but also grown for the production of alcohol, bioethanol and fuel.

**FIGURE 1:**
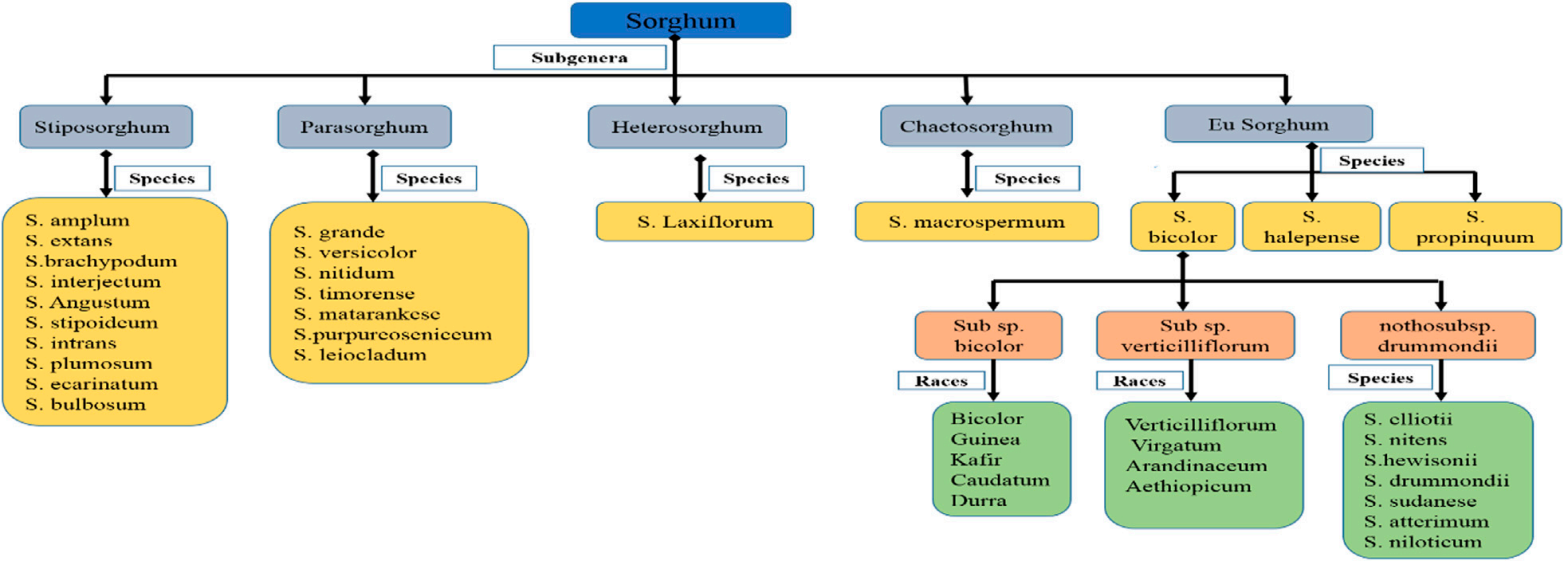
Sorghum classification (Wiersema and Dahlberg, 2007; U.S. Department of Agriculture, 2020).

The *Sorghum* genus is separated into five taxonomic subgenera or sections: Eu-Sorghum, Chaetosorghum, Heterosorghum, Para-Sorghum and Stiposorghum (Figure 1) (Garber, 1950). The first one includes all cultivated varieties and landraces of *S. bicolor* subsp. *bicolor*, as well as the wild and weed species *S*. *halepense* (L.) Pers. (Johnsons grass), *S*. *propinquum* (Kunth) Hitchc, *S*. *almum* Parodi, *S*. *drummondii* (Steud.) Millsp. and Chase, and *S*. *arundinaceum* (Desv.) Stapf. (the known progenitor of *S*. *bicolor*) (de Wet and Harlan, 1971; Doggett, 1988). The other four sections contain 19 wild species native of Africa, Asia and Australia (Garber, 1950; Lazarides et al., 1991). The wild sorghum was first utilized as nourishment, 7,500 B.C. in the Sahara region (Venkateswaran et al., 2019). Traces of both cultivated and wild sorghum were found from 3,500–1,500 B.C. in fired clay of Kassala region (Beldados and Costantini, 2011; Beldados et al., 2015). The most primitive domesticated sorghum was found 4,000 B.C. in Sudan (Winchell et al., 2017). The location of sorghum domestication is ambiguous (Venkateswaran et al., 2019). From its origin, i.e., Africa, domesticated sorghum begun dispersion via different means, like trade routes. Human migration brought sorghum from East to Southern Africa (Mann et al., 1983). Sorghum reached India through the trade routes of the Middle East (Mann et al., 1983). From India, it reached China through land and sea routes. However, the Indo-China river valley is regarded as an important route for this sorghum journey (Venkateswaran et al., 2019). In the 19th century, sorghum was introduced to the United States of America (USA) from China by traders, and it was then brought to Australia by Americans in the 1900s (Venkateswaran et al., 2014).

Sorghum is cultivated across the globe in almost 110 countries, mainly in Africa and Asia but also in America, Europe and Oceania (Popescu et al., 2018). The plant up to 2 m high, is mostly grown in regions with low rainfall and high temperatures (Mundia et al., 2019). Sorghum can grow with few water supply, and is often considered as a drought-tolerant crop. Moreover, it can tolerate elevated temperatures in comparison to other cereals (Hall, 2000), typically temperatures in the range 24–27°C after germination (Mundia et al., 2019). It is mostly grown in areas receiving annual precipitation between 350 and 700 mm. Sorghum needs very low fertilization and minimum pesticide treatment, and can be cultivated in marginal soils.

Sorghum is genetically and taxonomically related to maize (Taylor, 2019). Both are diploid plants with 10 chromosomes (2n = 20) (Obilana, 2004). However, the genome of sorghum is relatively smaller than that of maize, with a value of ca. 730 (Paterson et al., 2009) and ca. 2,300 Mbp (Megabase pairs) (Schnable et al., 2009), respectively.

**FIGURE 2:**
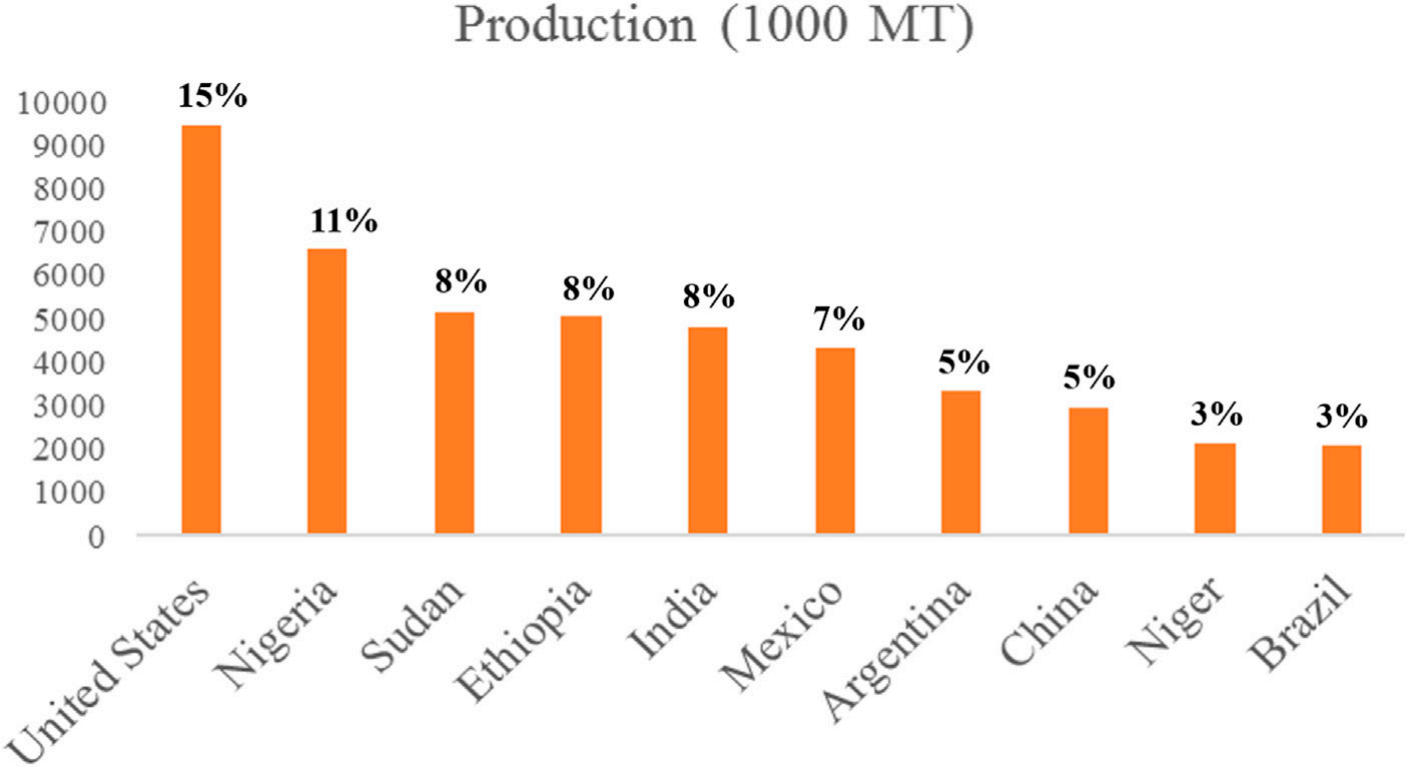
Production statistics and percentage share of the world’s top 10 sorghum-producing countries (U.S. Department of Agriculture, 2020).

According to the U.S. Department of Agriculture (2020), total global sorghum production was 62,641,000 MT. Nigeria, Sudan, and Ethiopia were the leading countries in the world regarding sorghum production (Figure 2). United States of America (USA) (7085,000 MT), Argentina (2241,000 MT), Australia (1678,000 MT), Kenya (74,000 MT), and Ukraine (59,000 MT) were the top five sorghum exporting countries (U.S. Department of Agriculture, 2020). It has several names in various regions of the world, including Jowar (India), Dura (Sudan), Mtama (Eastern Africa), Kaoliang (China), milo (USA), and Kafir corn (South Africa) (Mohapatra et al., 2017).

### Physiological response of sorghum to abiotic stresses

Sorghum is a crop that is well-adapted to abiotic stresses including drought, salinity, heat, and cold stress (Behera et al., 2022). When sorghum is exposed to abiotic stress, it triggers a series of physiological responses to help the plant adapt to the stress and survive. Some of the common physiological responses of sorghum under abiotic stress are 1. Osmotic Adjustment: Sorghum plants under drought stress start to accumulate solutes such as amino acids, proline, and sugars in their cells, which help maintain cell turgor pressure, and thus, avoid wilting 2. Reduced transpiration rate: During drought stress, sorghum plants can reduce their transpiration rate by closing their stomata to conserve water. This is achieved through the production of abscisic acid (ABA), which triggers stomatal closure 3. Alteration of root architecture: Sorghum plants exposed to drought stress alter their root system to explore deeper soil layers where water is more abundant. This involves the elongation of the main root and an increase in lateral roots 4. Changes in photosynthesis: Sorghum plants reduce their photosynthetic activity when exposed to abiotic stress in order to conserve water. This can lead to a reduction in biomass production and yield 5. Increased antioxidant activity: Sorghum plants increase the production of antioxidant enzymes such as catalase, superoxide dismutase, and peroxidase to detoxify harmful reactive oxygen species (ROS) that accumulate under abiotic stress 6. Accumulation of compatible solutes: Sorghum plants can accumulate compatible solutes such as betaine, trehalose, and proline in response to salt stress. These solutes help maintain osmotic balance and protect cellular structures from damage.

### Drought stress

Plant response to drought stress and drought tolerance is a result of complex biological processes involving physiological, biochemical, genomic, proteomic and metabolomics changes (Ngara et al., 2021). Stress-induced physiological changes, such as a shift in photosynthetic rate, were seen in genotypes of drought-sensitive sorghum (Fracasso et al., 2016). Photosynthetic rate, transpiration rate, water usage efficiency (WUE), and stomatal conductance are all significantly impacted by drought stress. An essential metric for assessing how drought stress affects photosynthesis is the Fv/Fm, which stands for maximum quantum yield of photosystem II (Husen et al., 2014). It serves as a measure of photosynthetic efficiency and is much lower in sorghum cultivated under drought stress circumstances (Johnson et al., 2014). According to Zhang et al. (2019), drought stress reduces stomatal conductance and transpiration rate, increases quantum yield and leaf temperature, decreases chlorophyll and Rubisco, increases O_2_ evolution, and decreases PEPCase activity in sorghum (Bao et al., 2016). Many studies have demonstrated that drought-tolerant sorghum genotypes have noticeably higher Fv/Fm and photosynthetic rate values (Fracasso et al., 2016; Sukumaran et al., 2016). The photosynthetic recovery that occurs after rehydration has a significant part in defining plants’ tolerance to drought as well as in preventing a decrease in grain yield, in addition to their capacity to avoid and/or tolerate drought stress (Chaves et al., 2009).

Transpiration efficiency in drought-tolerant sorghum genotypes did not differ between control and drought stressed plants, but in drought sensitive genotypes, there was a statistically significant difference between control and drought stressed plants (Fracasso et al., 2016). In addition, during the drought stress period, genotypes that were more resistant to drought displayed a much higher WUE than those that were more susceptible to it (Fracasso et al., 2016). In sorghum, it has been found that grain yield is highly correlated with water extraction and transpiration efficiency (Vadez et al., 2011b). Drought resistant genotypes are those that preserve water that can be used during the grain filling stage in water-limited areas and have lower stomatal conductance and transpiration rates during the vegetative phase (Lopez et al., 2017). According to a study by Lopez et al. (2017), the QTL for stomatal conductance was linked to lower transpiration rates but not to shoot biomass or photosynthetic rates.

A plant’s capacity to maintain a normal level of chlorophyll under drought stress factors into its capacity to respond to drought (Chen et al., 2016). The ability of the plant to absorb light for photosynthesis is directly influenced by the amount of total chlorophyll as well as chlorophyll a and b. Several investigations have found a considerable decrease in the amount of chlorophyll in sorghum cultivated under drought stress (Rama Reddy et al., 2014; Fracasso et al., 2016; Amoah and Antwi-Berko, 2020). When compared to equivalent genotypes grown under control conditions, the total chlorophyll content of stay-green genotypes underwent a 23% decline while senescent genotypes underwent a 75% reduction (Xu et al., 2000). According to another study, stressed plants had 4.3% less total chlorophyll than control plants (Devnarain et al., 2016). Together with chlorophyll, lower concentrations of certain carotenoids have also been seen under extreme drought stress circumstances (Munné-Bosch et al., 2001). According to Takele (2010), drought-tolerant sorghum’s pre- and post-flowering stages have lower chlorophyll and carotenoid concentrations. The downregulation of genes involved in the terpenoid and carotenoid biosynthesis in drought sensitive genotypes is likely the cause of a drop in carotenoids (Fracasso et al., 2016). Drought stress causes downregulation of genes involved in the biosynthesis of carotenoids and chlorophyll, which has a significant impact on the pathways for light reaction and carbon fixation. The number of green leaves and the area of green leaves at the blooming and maturity stages were significantly positively correlated with the chlorophyll concentration at maturity. In turn, grain yield highly linked with both leaf characteristics at both stages (Rama Reddy et al., 2014).

Loss of chlorophyll and a steady decline in photosynthetic capacity are the characteristics of leaf senescence (Borrell et al., 2000; Tao et al., 2000). Stay-green is a well-known feature that helps sorghum adapt to post-flowering dry conditions by delaying leaf senescence and enhancing grain output. Several researchers have worked to explain the physiological basis for sorghum’s ability to stay green. Early theories and research showed that stay green under drought stress conditions is linked to greater leaf nitrogen concentrations, cytokinin levels, and chlorophyll content. As an illustration, Borrell et al. (2000) discovered a link between stay green and greater leaf nitrogen concentrations, particularly during the flowering stage. A further study found that the stay-green sorghum genotypes maintain high amounts of cytokinin, which suggests a slower rate of senescence (Thomas and Howarth, 2000). Moreover, compared to senescent genotypes, the stay-green genotypes exhibit higher amounts of chlorophyll concentration (Xu et al., 2000).

The stay green trait of sorghum as a response to drought is associated with higher leaf chlorophyll content, slower rate of loss of green leaf area (Kassahun et al., 2010), decreased tillering and size of upper leaves (Borrell et al., 2014a; Borrell et al., 2014b; George-Jaeggli et al., 2017). Moreover, the characteristic is associated with better water extraction and transpiration efficiency (TE) (Vadez et al., 2011a). The introgression of stay green QTLs from B35 to senescent variety R 16 under drought stress conditions resulted in higher leaf chlorophyll levels at flowering and a greater percentage of green leaf area during grain filling stages, which were correlated with a higher relative grain yield during the post-flowering stages (Kassahun et al., 2010). At blooming phases, the Stg QTL regulates canopy size by reducing tillering, increasing the size of top leaves, reducing the size of lower leaves, and in some situations, reducing the number of leaves per culm. The reduced canopy size at flowering decreases pre-flowering water demand, thereby leads to increased water availability during grain filling stage, which in turn lead to increased biomass production and grain yield (Borrell et al., 2014b). Old leaves are shed at the flowering stage as a result of lower leaves’ accelerated age-related senescence in stay-green lines, which results in a smaller canopy (George-Jaeggli et al., 2017). Any pre-flowering water savings boost post-flowering water availability, allowing plants to maintain their photosynthetic potential for a longer length of time and “remain green” throughout grain filling (George-Jaeggli et al., 2017). Remain green may be viewed as a post-flowering drought-tolerance mechanism that makes it easier to get the water needed for both general growth and grain production.

### Aluminium stress

Plant production in acidic soils is greatly limited by the rhizotoxicity of Al3+. This problem is worsened by the use of ammonium fertilizers and acid rain. The concentration of Al3+ in the soil solution is higher at pH 3.7 compared to pH 5.8 or 6.3, as reported by Miller et al. (2009). The resistance mechanisms employed by plants against Al3+ toxicity can be categorized into two groups: external tolerance, involving the chelation of the metal ion by organic acids in the rhizosphere, and internal tolerance, involving the chelation of Al3+ within the cells (Inostroza-Blancheteau et al., 2008). Sorghum bicolor, a plant belonging to the first group, uses the exclusion mechanism to alleviate the toxicity of Al3+. This involves the release of organic acid exudates from root cells to the apoplast, which can bind and detoxify the harmful Al3+ cations outside the cell. Magalhaes et al. (2004) identified a major locus (Altsb) for Al3+ tolerance in two sorghum cultivars, which may be linked to citrate exudation from root apices. Furthermore, a gene encoding an aluminum-activated citrate transporter, a member of the multidrug and toxic compound extrusion (MATE) family, has been discovered in sorghum plants (Magalhaes et al., 2007). It is worth noting that genes controlling Al3+ resistance have been cloned from different crop plants, including sorghum (Ryan and Delhaize, 2010).

### Waterlogging

In regions with tropical and sub-tropical climates, heavy rains, storms, excess irrigation or flooding may cause intermittent or long-term waterlogging of crops. This can be particularly detrimental to the crops as it not only affects their metabolism but also causes undesirable changes to the soil texture. A study by Promkhambu et al. (2010) compared the responses of three sweet sorghums and a forage cultivar after 20 days of waterlogging. The long-term flooding resulted in a significant reduction in biomass production, increased allocation of biomass to the roots, and a decrease in leaf area. In the sweet cultivars, it also significantly reduced photosynthetic rate, stomatal conductance, and transpiration. One sweet cultivar, Wray, showed high tolerance to waterlogging as evidenced by its ability to extend youngest leaves, produce new leaves, increase root length, and nodal root development. The adaptation of plants to O2 deficiency is based on their ability to maintain active fermentative metabolism under anaerobiosis. A flood-tolerant sorghum cv. SSG-59-3 exhibited a constant increase in alcohol dehydrogenase (EC 1.1.1.1) and lactate dehydrogenase (EC 1.1.1.27) activities, as well as higher ethanol concentration than the sensitive variety S-308. This suggests that the flood-tolerant variety tends to attain greater capacity for various fermentative pathways as alternative means to sustain production of ATP under flooded conditions (Jain et al., 2010).

### Salt stress

Although sorghum is a salt-tolerant crop, there are genotypic variances among cultivars. High salinity is caused by an excess buildup of several ions in the soil, primarily sodium, calcium, magnesium, chloride, and sulphate, with sodium chloride being the most detrimental to plant growth and development. After 4 days of growth in 200 mM NaCl, a substantial salt-stress phenotype was found in sorghum plants (Kumar Swami et al., 2011). In sweet sorghum, salt stress decreased the percentage and lengthened the duration of germination (Gill et al., 2003; Almodares et al., 2007). Substantial variability in germination susceptibility to high salinity can be found among cultivars. Toxic ion accumulation (Na+ and Cl) disrupts ion uptake and K+ status of tissues; hence, high K+/Na + discrimination and the regulation of a low Na+/K+ ratio in tissues characterize salt-tolerant genotypes (Hasegawa et al., 2000). The Na + content of tissues in sorghum rose with additional external sodium concentrations (de Lacerda et al., 2003), whereas root and shoot Na + contents differed significantly between genotypes (Bavei et al., 2011a). Reduced sodium buildup in the shoot is the result of either decreased Na + uptake by the root or variations in the rate of Na + transport to the shoot. Jambo, a salt-tolerant sorghum variety was reported to accumulate less Na+ in root and shoot tissues than salt-sensitive genotypes and maintain lower Na+/K+ ratios in both root and shoot tissues (Bavei et al., 2011a). The salt-tolerant genotype Jambo was also shown to accumulate more Ca2+ in both leaf and root tissues than the sensitive types, Kimia and Payam (Bavei et al., 2011b). Several studies have linked salt tolerance to an increase in antioxidant enzyme activity. However, greater antioxidant activities did not consistently depend on salt tolerance, and precise tuning of both enzymatic and non-enzymatic ROS-scavenging constituents can contribute to successful acclimatization. Silicon application to soil mitigated salinity stress in two sorghum cultivars and resulted in an increase in the activities of ascorbate peroxidase, catalase, superoxide dismutase, peroxidase, glutathione reductase and total phenol and antioxidant contents of tissues, indicating that the mitigation of salinity stress was associated with increased antioxidant activity. In addition, after silicon treatment, the plants accumulated compatible osmolytes, soluble carbohydrates, and proline and displayed greater osmotic adaptability (Kafi et al., 2011). Stem yield and soluble carbohydrate levels declined with increasing salt in two sweet sorghums (cvs Keller and Sofra) and one grain sorghum cultivar (Kimia), however at the highest salinity level, cv. Keller had the greatest stem yield and sucrose content (Almodares et al., 2008). Although various authors have published analyses of transcriptomes in response to abiotic stressors (Dugas et al., 2011), investigations on the cellular proteome in sorghum are scarce. Swami et al. (2011) examined at how the protein complement of sorghum leaves changed after 96 h of exposure to 200 mM NaCl. They demonstrated 21 areas with changed expressions on 2-DE gels and identified them using a MALDI-TOF/TOF mass spectrometer after tryptic digestion of the excised spots. One protein, ATP synthase a-subunit, showed increased abundance, indicating that salt stress had an effect on the photosynthetic machinery. Eight of the upregulated proteins were engaged in scavenging reactive oxygen species (POX and APX) or detoxifying reactive electrophilic chemicals (glutathione S-transferase, EC 2.5.1.18). Additional proteins that may be involved in Na + -induced signal transduction include lectin-like protein kinase, salt-inducible protein kinase, and serine/threonine protein kinase.

### High and low temperature stresses

The total sugar content and biomass output of sweet sorghum are determined by the planting date: the later the planting, the lower the stalk yields in arid conditions (Almodares and Mostafafi Darany, 2006). In hot and dry climate zones, the key criteria determining planting date are soil water scarcity and cultivar heat stress sensitivity (Teetor et al., 2011). During a delayed harvest period, a reduction in carbohydrate components in the stems of Chinese sweet sorghum cultivars were reported (Zhao et al., 2012). Chilling stress in early spring can limit optimal development in temperate zones, determining the planting date in these regions. Sweet sorghum is a cold-sensitive crop, and low temperatures affect seed germination, seedling emergence, and plant growth. Burow et al. (2011) established simple sequence repeat (SSR) molecular markers related with several features for early-season cold tolerance in order to generate elite sorghum lines with stable and good early-season cold resistance. The mapping population is comprised of 171 F7-F8 recombinant inbred lines (RILs) generated from the cross of cold-sensitive RTX430 and cold-tolerant PI610727 lines. Gaigao Liang is another name for PI610727, a landrace from Chinese germplasm selected for early-season cold resistance. The RILs were tested in the laboratory for cold and ideal temperature germinability, field emergence, and seedling vigour in two locations during early-season planting. All traits had two or more quantitative trait loci (QTLs), except seedling vigour, which had only one QTL. The authors labeled the genomic areas of sorghum that have significant contributions to traits for early-season cold tolerance using PI610727, a new source of cold tolerance. Extreme temperature stress may also lead to a decrease in biomass and sugar output. Heat stress has a substantial impact on photosynthetic activity, light reactions, and the activity of Calvin cycle enzymes (Yan et al., 2011). It has been revealed that photosynthetic activity is affected not only by daytime temperatures but also by nighttime temperatures (Prasad and Djanaguiraman, 2011). The authors examined the effect of an optimal day/night temperature combination (32/22 °C, respectively) to that of an optimal day temperature (32 °C)/high night temperature (HNT) (28 °C) combination and observed that HNT negatively impacted the photosynthetic activity of plants. Exposure to HNT enhanced thylakoid membrane damage and non-photochemical quenching, but lowered chlorophyll content of tissues, photochemical quenching parameter, electron transport rate, and photosynthetic activity of leaves. In addition, HNT boosted the generation of ROS in leaves and pollen grains and led to decreased pollen germination and lower seed set. Prasad et al. (2011) also found that grain sorghum pollen had shorter lifespan and showed much lower germination percentage on artificial growth medium at higher temperatures. Moreover, when compared to control plants grown at ideal temperatures (32/22°C), high temperature stress (40/30°C day/night temperatures) decreased chlorophyll content, photosynthetic rate, and antioxidant enzyme activities while increasing oxidant generation and membrane damage. Heat shock proteins (HSPs) are proteins that are produced in reaction to high temperatures or other abiotic stresses, and as molecular chaperones, they can protect proteins from the stressors’ damaging effects. The expression of hsp90 was compared in different types of sorghum (grain and forage sorghum hybrids, as well as in sweet sorghum cultivar) following varied heat stress durations (Pavli et al., 2011). The accumulation of hsp90 transcripts was measured by RT-qPCR analysis, and the levels of gene expression were shown to be significantly different in the genotypes studied. Sorghum’s whole genome sequence has been published. In combination with the investigation of abiotic stress-induced transcriptomes, proteomes, and metabolomes, this gives a valuable tool for breeders looking to improve the stress tolerance of this vital energy crop.

### Genetic resources of sorghum

The two most important sorghum germplasm banks are the International Crops Research Institute for the Semi-Arid Tropics (ICRISAT, India) and the United States Department of Agriculture’s National Plant Germplasm System (USDA-NPGS). Each house upwards of 41,000 sorghum accessions, along with historical accessions, landraces, wild relatives, and breeding lines.

The National Bureau of Plant Genetic Resources in India has about 20,000 collections. The greatest collection of Australian wild sorghums is held by the Australian Tropical Crops and Forages Genetic Resources Center. While in China, the Institute of Crop Germplasm Resources is holding over 16,874 collections. Sorghum was domesticated and evolved in North-east Africa, a unique that extend from Sudan to Ethiopia (Wendorf et al., 1992). As a result, the oldest wild types and greatest diversity can be found in Ethiopia and Sudan.

The largest collection of accessions (almost 41,000) kept at the USDA-NPGS having 7, 217 accessions from Ethiopia, and 2,552 were from Sudan as reported by Dahlberg et al. (2004). Although these two regions are the principal source of sorghum origin, their germplasm resources differ substantially. Five ancestral populations make up a representative Sudan core collection as opposed to eleven ancestral populations in the Ethiopia core set. Additionally, the pairwise genetic distance between the accessions in the Sudan core set is greater than it is in Ethiopia, indicating that Sudan collection has a greater genetic variability (Cuevas and Prom, 2020). Major gene bank collections are enlisted in Table 1.

**TABLE 1:** Sorghum germplasm conserved in major gene banks.

Region/Country	Institute/Organization	Wild	Cultivated	Total (% of total)
Africa				
Ethiopia	Institute of Biodiversity Conservation (IBC)		9772	9772 (4.1)
Kenya	National GeneBank of Kenya, Crop Plant Genetic Resources Centred Muguga (KARI-NGBK)	92	5774	5866 (2.5)
Zambia	SADC Plant Genetic Resources Centre (SRGB)	27	3692	3719 (1.6)
America				
The United States	Plant Genetic Resources Conservation Unit, southern Regional	199	43,511	43,710 (18.5)
	Station Plant Introduction Station, University of Georgia, National			
	Centre for Genetic Resources Preservation			
Brazil	Embrapa Milo et Sorgho (CNPMS), Embrapa Recursos Geneticos e		10,812	10,812 (4.6)
	Biotechnologia (CENARGEN)			
Mexico	Programa de Recursos Geneticos, Centro de Investigaciones		5500	5500 (2.3)
	Forestales y Agropecuarias (CIFAP-MEX), Estacion de Iguala,			
	Instituto Nacionale de Investigaciones Agricolas (INIA-Iguala)			
Asia				
India	International Crops Research Institute for the Semi-Arid Tropics	461	39,092	39,553 (16.8)
	(ICRISAT)			
	ICAR National Bureau of Plant Genetic Resources	11	20,555	20,566
India	Indian Institute of Millets Research	27	23,059	23,086
China	Institute of Crop Science, Chinese Academy of Agricultural Sciences		18,263	18,263 (7.7)
	(ICS-CAAS)			
Japan	Department of Genetic Resources National Institute of	13	5061	5074 (2.0)
	Agrobiological Sciences (NIAS)			
Pakistan	Plant Genetic Resources Programme (PGRP)	16	1716	1732 (0.7)
Europe				
Russian Federation	NI Vavilov All-Russian Scientific Research institute of Plant Industry		3963	3963 (1.7)
	(VIR)			
France	Laboratoire des Resources Genetiques et Amelioration des Plantes	27	7278	7305 (3.0)
	Tropicales, ORSTOM, Centre de Cooperation Internationale en			
	Recherche Agronomique pour le Developpement (CIRAD),			
Australia				
Australia	Australian Tropical Crops and Forages Genetic Resources Centre	346	4144	4491 (2)
	(ATCFA)			

Source: (Aruna et al., 2018).

## Sorghum genomic resources

### Structural genomics: molecular markers (from hybridization based to sequenced based) in sorghum

The Restriction Fragment Length Polymorphism (RFLP) marker system has been initially widely used in sorghum (Tao et al., 1998). Thereafter, more marker systems emerged for sorghum, including Amplified Fragment Length Polymorphism (AFLP), Randomly Amplified Polymorphic DNA (RAPD), Single Sequence Repeats (SSRs), and Diversity Array Technology (DArT) markers (Haussmann et al., 2002; Bowers et al., 2003; Wu et al., 2006; Mace et al., 2008). Each of these marker systems was utilized in sorghum for various objectives, including QTL mapping, genetic diversity analysis, and fingerprinting. The genome sequence accessibility of sorghum offers a chance to widen the embankment of SSRs molecular markers. This genome sequence predicts around 71,000 SSRs. The sorghum complete genome sequence and Next-Generation Sequencing (NGS) approaches like genotyping-by-sequencing (GBS) made it possible to speed up the production of valuable and cheap maker systems.

Publication of initial reference genome accessions for sorghum (Paterson et al., 2009), together with advances in NGS tools and speedy progress in Computational Tools (CTs), have created innovative opportunities for the swift and genomic DNA markers applicable for high-throughput genotyping (HTG) in sorghum. The recent improvements in sequencing and complete-genome genotyping tools, including NGS and Whole-genome marker arrays (WGMA), allowed the assembly of extensive genomic resources conservation in sorghum (Morris et al., 2013a; McCormick et al., 2018). The NGS technique, including Whole genome shotgun technique (WGST) and Illumina Genome Analyzer sequencing technology (IGAST), together with more swift placement software, utilized to re-sequence accessory sorghum lines to explore a wide number of structural variants and SNPs in formerly sequenced genomes of sorghum (Nelson et al., 2011; Morris et al., 2013b; Bekele et al., 2013). SNPs, copy number variations, in-dels (insertions-deletions), duplications, translocations, inversions, and absence/presence variants are all types of structural changes that can happen in a genome (Saxena et al., 2014).

GBS is a commonly utilized genotyping technique in GWAS that combines multiplex to detect high-density SNPs with reduction enzymes for minimizing genome convolution (Elshire et al., 2011). Worthwhile quantity of sorghum accessions has been sequenced using the GBS tool to evaluate the genetic diversity (GD) and structure (Morris et al., 2013a). Determining the GD in sorghum germplasm collections facilitates the detection of narrative alleles and genes linked with crucial characters and also used to improve the germplasm in breeding programs. Multiple genomic resources are available for the sorghum research community, comprising DNA markers like SSR, DArT, SNPs (Billot et al., 2012; Evans et al., 2013), the high-density genetic maps (Kong et al., 2013), and sequenced genomes (Mace et al., 2013). Sorghum’s genome has been thoroughly assembled and annotated, making it possible to use RAD-seq (Restriction site-associated DNA tags sequencing), which are dispersed widely across the genome. High-resolution genotyping (HRG) and SNP identification is dependent on a decrease in genomic complexity. Nelson et al. (2011) identified 283,000 numbers of SNPs from 8 accessions of sorghum using a RAD-seq sequencing approach. Changes in the structure of genomes are a key part of biological and evolutionary processes, and research must be done to find a link between genetic differences and how well plants do.

### Progress towards the development of trait-specific recombinant inbred line mapping populations

Several minor genes govern quantitative features of agronomic concern, which are heavily impacted by the environment. Therefore, in order to determine genes and QTLs that interact with their surroundings, genetic examination of these traits needs growth to occur over a period of time that comprise numerous years and venues (Kumar et al., 2018). In parallel with the focus that has been placed on the development of the mapping populations, significant real progress has been made around the world in the quest to create trait-specific RILs (recombinant inbred line) for key traits in sorghum, such as earliness, drought resilience, root traits, early growth vigor, biotic resistance and Zn and Fe content (Tuinstra et al., 1996; Kotla et al., 2019). The 342 RIL (F6) populations were developed from cross 296B x PVK 801 to check Fe and Zn concentrations and to create linkage maps and QTLs detection for Zn and Fe (Kotla et al., 2019).

Drought is one of the most deleterious factors affecting agricultural productivity in tropical regions, along with other abiotic factors. “Terminal drought” is used to describe drought that occurs during the grain-filling stage. “Stay green” is one of the features that is considered to be responsible for a plant’s ability to withstand extreme drought. Researchers from a variety of institutions have discovered QTLs that are responsible for resilience to the terminal drought (Haussmann et al., 2002; Harris et al., 2007), whereas Crasta et al. (1999) identified 2 QTLs for maturity and 7 QTLs for terminal drought tolerance (Table 2). Within these 7 QTLs, 3 were contributed around 42% of the phenotypic variability, while 4 minor QTLs accounted a combined 25% of the phenotypic diversity in terms of stay green assessments. Only 4 significant QTLs were reported in sorghum to be responsible for the stay green characteristic (Sanchez et al., 2002; Harris et al. (2007). B35 genotype was used as donor parent in both of the tests that were conducted. The E36-1 genotype served as the parent material in a further investigation that aimed to determine the QTLs that control the stay green traits in sorghum (Haussmann et al., 2002). By producing 245 F9 RILs resulting from a hybrid between M35-1 and B35, Nagaraja Reddy et al. (2013) were able to identify 61 QTL for a variety of different markers of stay green characteristics. Their expression featured a significant amount of Stg2, Stg3, and StgB.

**TABLE 2:** Summary of numbers of stay green QTLs identified by developing RILs.

Sr. No.	Parents population	Population type	Population size	No. of QTLs	Studies
1	E36-1 × N13	RILs	226	21	Haussmann et al. (2002)
2	SC283 × BR007	RILs	100	4	Sabadin et al. (2012)
3	E36-1 × IS9830	RILs	226	19	Haussmann et al. (2002)
4	Tx7000× SC56	RILs	125	14	Kebede et al. (2001)
5	Tx7000 × B35	RILs	98	4	Subudhi et al. (2000)
6	Tx7000 × B35	RILs	98	3	Xu et al. (2000)
7	B35 × M35-1	RILs	245	43	Nagaraja Reddy et al. (2013)
8	IS18551 × 296B	RILs	168	9	Srinivas et al. (2009b)
9	QL39/QL41	RILs	152	5	Tao et al. (2000)
10	Tx430 × B35	RILs	96	7	Crasta et al. (1999)

## Development of sequenced based high-density map

### Early sorghum genetic maps

Mapping studies in sorghum-containing DNA markers were initiated at the beginning of the 1990s (Table 3). DNA fragments characterized in maize were found to hybridize strongly with sorghum DNA and many other additional crops, comprising Johnson grass, sugarcane, and foxtail millet signifying that polymorphic loci both in sorghum and maize are specific (Hulbert et al., 1990). Moreover, RFLPs probes derived from maize were utilized in sorghum to construct linkage map and it was discovered that the maize probes were similar to the sorghum probes (Whitkus et al., 1992; Berhan et al., 1993). This made it possible to investigate comparative genetics among species that are genetically linked to one another and opened the door for the possibility of evaluating horizontal transfer of genes from other species to sorghum simultaneously. In order to determine the synteny and homology shared by the various members of the Poaceae family, the earlier maps that contained both endogenous and exogenous RFLP probes proved to be very useful in comparative genomic research. In addition, probes obtained from cDNA and genomic DNA (gDNA) particular sorghum were incorporated into linkage maps alongside exogenous probes that were established from other related genomes. Ragab et al. (1994) created a map that consisted of 33 maize probes and 38 sorghum probes. The length of the map was 633 cM, and the average distance between markers was 8.9 cm. Subudhi and Nguyen (2000) integrated the maps of a RIL population to align five primary RFLP maps with 10 linkage groups. This was done by aligning the maps using linkage groups. This assisted us in determining how QTL markers located in various sections of the genome are connected to one another and how accurate the maps that we currently possess are.

**TABLE 3:** Sorghum genetic linkage maps.

Parents	Population	Marker	Map length (cM)	Linkage group	Reference
IS 18809 × IS 2482C	(F2) 81	91 RFLPs, 7 isozymes	949	13	Whitkus et al. (1992)
249 × IS 2807	91 RIL (F5)	133 RFLPs	877	13	Dufour et al. (1997)
CK 60 × PI 229828	78 F2	201 RFLPs	1,530	10	Pereira et al. (1994)
BTx 623 × BSC 35	93 F2:3	71 RFLPs	633	15	Ragab et al. (1994)
IS 24756 × IS 18729	(F2) 55	96 RFLPs	709	15	Berhan et al. (1993)
BTx 623 × Sorghum propinquum	56 F2	276 RFLPs	1,445	10	Chittenden et al. (1994)
379 × IS 2807	110 F5 RIL	145 RFLPs	977		Dufour et al. (1997)
IS 24756 × IS 18729	(F2) 149	35 RFLPs	440	5	Binelli et al. (1992)
PI 229828 × CK 60	152 F2	111 RFLPs	1,299	10	Pereira and Lee (1995)
TX 7078 × B 35	98 F5:7–8 RIL	20 RFLPs, 150 RAPD	1,580	17	Tuinstra et al. (1997)
IS 3620C × BTx 623	50 F2	190 RFLPs	1,789	14	Xu et al. (1994)
M 91051 × Shanqui Red	(F2) 55	37 RFLPs	283	8	Hulbert et al. (1990)
BTx 623 × S. propinquum	370 F2	202 RFLPs	935	11	Lin et al. (1995)
Composite map		183 RFLPs	1,096	12	Dufour et al. (1997)
CK 60 × PI 229828	68 F2	7 SSRs	1,575		Taramino et al. (1997)
QL 39 × QL 41	128 F5 RIL	155 RFLPs, 8 SSRs	1,400	21	Tao et al. (1998)
IS 3620C × BTx 623	137 RIL (F6)	344 RFLPs	1,364	10	Peng et al. (1999)
Tx 7000 × B 35	98 RIL (F7)	3 SSRs, 214 RFLPs and 7 RAPD	1,200	10	Subudhi and Nguyen (2000)
IS 2807 × 379	110 F5 RIL	298 RFLPs, 137 AFLPs	1,899	11	Boivin et al. (1999)
BTx 623 × IS 3620C	138 F6–8 RIL	114 RFLPs, 31 SSRs	1,287	10	Kong et al. (2000)
QL 41 × QL 39	152 RIL (F5)	281 RFLPs, 5 morphological markers, 25 SSRs	>2,750	10	Tao et al. (2000)
Tx 7000 × B 35	98 RIL (F7)	162 RFLPs	837	10	Xu et al. (2000)
B 35 × Tx 430	96 RIL (F5)	142 RFLPs	1,602	14	Crasta et al. (1999)
BTx 623 × IS 3620C	139 F6–8 RIL	323 RFLPs, 143 SSRs	1,406	10	Bhattramakki et al. (2000)
B2 V4 × 1383–2	150 F2	122 MSAP, 22 SSRs	483.6	11	Duan et al. (2009)
296B × IS 18551	168 RIL	38 EST-SSRs, 107 SSRs, 3 morphological markers, 10 Unigene SSRs	1,143	16	Satish et al. (2009)
Mixed map		154 RFLPs, 10 morphological markers, 34 SSRs,	1,450	10	Bennetzen et al. (2001)
SC 56 × Tx 7000	125 F7 RIL	144 RFLPs	1,355	10	Kebede et al. (2001)
N 13 × E 36-1	94 RIL	14 RFLPs, 45 genomic SSRs, 3 RAPD markers, 125 AFLPs, 55 EST-SSRs,	2,838	10	Ramu et al. (2009)
296B × IS 18551	168 RIL	100 SSRs, 28 EST-SSRs	1,074.50	15	Srinivas et al. (2009a)
296B × IS 18551	168 RIL	100 SSRs, 38 EST-SSRs, 10 Unigene SSRs, 2 morphological markers	1,098.50	15	Srinivas et al. (2009b)
BTx 623 × IS 3620C	137 F6–8 RIL	morphological markers, 259 RFLPs, 226 SSRs, 303 DArTs	1,528	10	Mace et al. (2008)
R 890592 × ICSV 745	119 RIL	234 DArTs, 244 RFLPs, 10 SSRs,	1,433	10	
IS 8525 × R 931945-2-2	RIL 146	47 SSRs, 357 DArTs, 188 AFLPs,	1,453	10	
SC 170-6-8 × B 923296	RIL 88	13 SSRs and 170 DArTs,	1,138	10	
QL 12 × BTx 642	RIL 94	117 DArTs	910	10	
SSM 249× SAR 10	183 RIL	131 SSRs, 627 DArTs, 47 RFLPs,	1,227	10	
Consensus map		839, Non-DArT markers and 1190 DArTs,	1,603.50	10	
IS 2122 × 27B	210 RIL	127 SSRs, one morphological marker and 21 genic-SSRs, and	700	10	Aruna et al. (2011)
M35-1 × B 35	245 RIL	228 SSRs and 3 morphological markers	1,235.50	10	Nagaraja Reddy et al. (2012)
Shihong 137 × L-Tian	186 F2	118 SSRs	1,884.60	15	Guan et al. (2011)
BR 007 × SC 283	90 RIL	255 DArTs, one RFLP marker 83 SSRs, and 5 sequence- tagged site (STS),	2,034.90	10	Sabadin et al. (2012)
SS 79 × M7 1	188 RIL	102 AFLP, 6 EST-SSR and 49 SSR,	1,029	11	Shiringani and Friedt (2011)
B 923296 × SC 170-6-8	141 RIL	377 DArTs	2,259	10	Mace and Jordan (2011)
BTx 623 × Sorghum propinquum	163 RIL	141 SSRs	773.1	10	Kong et al. (2013)
74LH 3213 × MS 138B	133 RIL	247 SSRs	1,697.20	10	Takai et al. (2012)
654 × LTR 108	244 RIL	3418 bin markers, SNPs	1,591.40	10	Zou et al. (2012)
M 71 × SS 79	188 RIL	6 EST-SSR, 49 SSR, 102 AFLP	1,029	11	Shiringani et al. (2010)
ICSV 745 × R 890562	119 RIL	234 DArTs, and 244 RFLPs	1,487.96	12	Alam et al. (2014)
296B × PVK 801	342 RIL	10 SSRs, 1148 DArTs, 927 DArTSeqs and 13 SSRs	1355.52		Kotla et al. (2019)
BT X 623 and NOG	213 RILs	3710 SNPs	1,299.70		Kajiya-Kanegae et al. (2020)
Tx623A and sudangrass sa	RIL	1,065 SLAF	1191.7		Jin et al. (2021)
		markers			

### High-density genetic maps with various types of markers

In sorghum, high-density genetic maps can be utilized for useful gene mining, genome comparison, and gene mapping. In addition, they improve the statistical power and accuracy of recognizing QTLs and genes (Ji et al., 2017). In contrast to other molecular markers, RAPD and RFLP molecular markers have been of narrow use in the constructing of sorghum genetic mapping. RAPD markers have a dominant pattern of heritage and inadequate repeatability. As a result, their application in sorghum genetic analysis is confined. Due to the accuracy of the AFLP method for genetic mapping in sorghum, it was frequently used in the saturation of linkage maps established previously with RAPD and RFLP markers. AFLP markers have been included on sorghum linkage maps so that map early sensory and QTL identifications can be performed (Shiringani and Friedt, 2011). Despite this, AFLPs is not widely used because of the complexity involved in map-based cloning application with highly marker-assisted breeding tools. Additionally, this technique requires a substantial amount of time to obtain results and is costly too. So, it was important to come up with quick and cheap ways to figure out a plant’s genotype.

For plant genotyping, SSRs markers with a dense level of polymorphism have become severely popular than AFLP and RAPD due to their ease of use, low cost, automation and throughput level (Varshney et al., 2005). Such markers have greatly provide the molecular inspection of agriculturally essential characters in various crops such as sorghum (Murray et al., 2008; Kumar et al., 2015). Bhattramakki et al. (2000) described a mixed RFLP and SSR linkage map of sorghum employing a significant number of RFLP and SSR loci of sorghum. This map was used to determine the genetic linkage of sorghum. The application of RAPD, RFLP, SSR and AFLP markers has been pervaded by less reproducibility and genome coverage. DArT markers have the capability of whole-genome profiling and high-throughput based hybridization technology. Sabadin et al. (2012) used a BR007 x SC283 cross to create a genetic map of sorghum that included 255 DArT markers, 1 RFLP, 83 SSRs, and 5 sequence-tagged sites (STS). These genetic maps have the potential to serve as sources for a variety of genetic research projects as well as well as the incorporation of DArT markers with other genetic resources. As of right now, the SNPs exposed by NGS are the most desirable option of markers for use in plant breeding and plant genetic research. This is due to the fact that they are present in such high numbers in nearly every population. Access to huge collections of SNPs continues to make it possible for genetic techniques such as population structure, association research, linkage mapping, map-based cloning, functional genomics and marker-assisted breeding (Kumar et al., 2012). It was proved that resequencing is effective in sorghum by developing an ultra-high-density linkage map employing 244 RILs of sorghum crosses and high-quality SNPs derived from low-coverage genomes. This map was used to demonstrate the usefulness of resequencing (Zou et al., 2012).

With the improvement in sequencing technology, SNPs from genome sequencing have been frequently used for QTL analysis and genetic mapping (Berkman et al., 2012). Restriction Site Associated DNA Sequencing (RAD-seq) is a technique of reduced-representation genome sequencing. This technology has a high throughput at an affordable cost and is easy to use (Kim et al., 2016). The RAD-seq technique has been extensively employed in genetic mapping. This technique was used to build an important genetic map from a RIL population between Sudangrass and sorghum Tx623A. This map has a complete length of 1191.7 cm and comprises 1,065 markers (Jin et al., 2021).

### Reference genome of sorghum

Sorghum, being a diploid crop, has a relatively small and non-duplicated genome. The whole reference genome sequence of BTx623 was first assembled by Sanger sequencing (Paterson et al., 2009) and has been updated thereafter by McCormick et al. (2018). It is characterized by 58.8% retrotransposons, 8.7% DNA transposons, and ∼34,129 annotated genes. On its genomic basis, numerous sequence variations have been found, including large genomic copy number variation (CNV), insertions and deletions (InDels), and the presence and absence (PAV) of BTx623 (Zheng et al., 2011; Mace et al., 2013). There is now a number of sorghum SNP databases and microarray datasets accessible (Shakoor et al., 2014; Luo et al., 2016; Hua et al., 2019). Other sorghum genome sequences are also reported as a result of the advancement of sequencing technologies. Bionano Genomics Direct Label and Stain (DLS) optical mapping and Oxford Nanopore sequencing technology have been used to create a chromosome-scale *de novo* assembly of the genome of a grain sorghum line Tx430 (Deschamps et al., 2018). Tx430 varies from BTx623 in that it has a shorter median length and more predicted genes—39,510 in total. The sweet sorghum Rio genome was subsequently constructed to better understand its sugar metabolism. Rio’s final genome was 729.4 Mb in size, with 35,476 predicted genes, 54 of which were specific to Rio. In order to understand the metabolism of sugar, the genome of Rio’s sweet sorghum was subsequently assembled at the chromosomal level. Each of these genome assemblies is limited to its respective accession and does not reflect the diversity of genes in this species. However, the diversity within a species cannot be captured by a single reference genome for all species and therefore requires a “pan-genome” to truly represent diversity within an organism (Bayer et al., 2020). A pan-genome of sorghum was constructed using an ongoing mapping and assembly method. Of the 354 entire genomes that were sequenced, 176 of the accessions had a coverage greater than 10X. In comparison to the reference assembly’s 708 Mbp size, the pan-overall genome’s size has grown by 20% (175 Mbp), to 883 Mbp (Ruperao et al., 2021). The great amount of genetic variety shown in the various species is likely to blame for this level of new sequence growth (Cuevas and Prom, 2020). The newly constructed pan-genome includes an average of 32,795 genes across all cultivars, with a core genome of 16,821 genes. More reference genomes are needed to fully understand the genetic basis of phenotypic diversity that emerged throughout sorghum domestication and development. The statistics of the reference genome of sorghum are presented in Table 4.

**TABLE 4:** Sorghum bicolor reference genome statistics.

	Version 1	Version 3
Number of pseudomolecules	10	10
Number of contigs	6929	2688
Scaffold sequence (Mbp)	659.2	683.6
Contig sequence (Mbp)	625.6	655.2
Scaffold N50 (Mbp)	64.3	68.7
Contig N50 (Mbp)	0.204	1.5
Scaffold L50	05	05
Coting L50	838	71
Unmapped sequence (Mbp)	71.9	20.2
Estimated error frequency rate	<1 per 10 kbp	<1 per 100 kbp

Source: (McCormick et al., 2018).

An appreciation genome sequence was produced and annotated in the research carried out by McCormick et al. (2018). This was accomplished by employing transcriptome data, deep sequencing, and genetic linkage analysis. The reference genome sequence order has been increased, an additional 29.6 Mbp of sequence has been incorporated, the quantity of genes that have been annotated has increased by 24%–34,211, the median gene length and N50 have both increased, and the inaccuracy frequency has decreased by a factor of ten to 1 per 100 kbp.

## Functional genomics toward the identification of candidate genes

### Marker assisted breeding in sorghum

**FIGURE 3:**
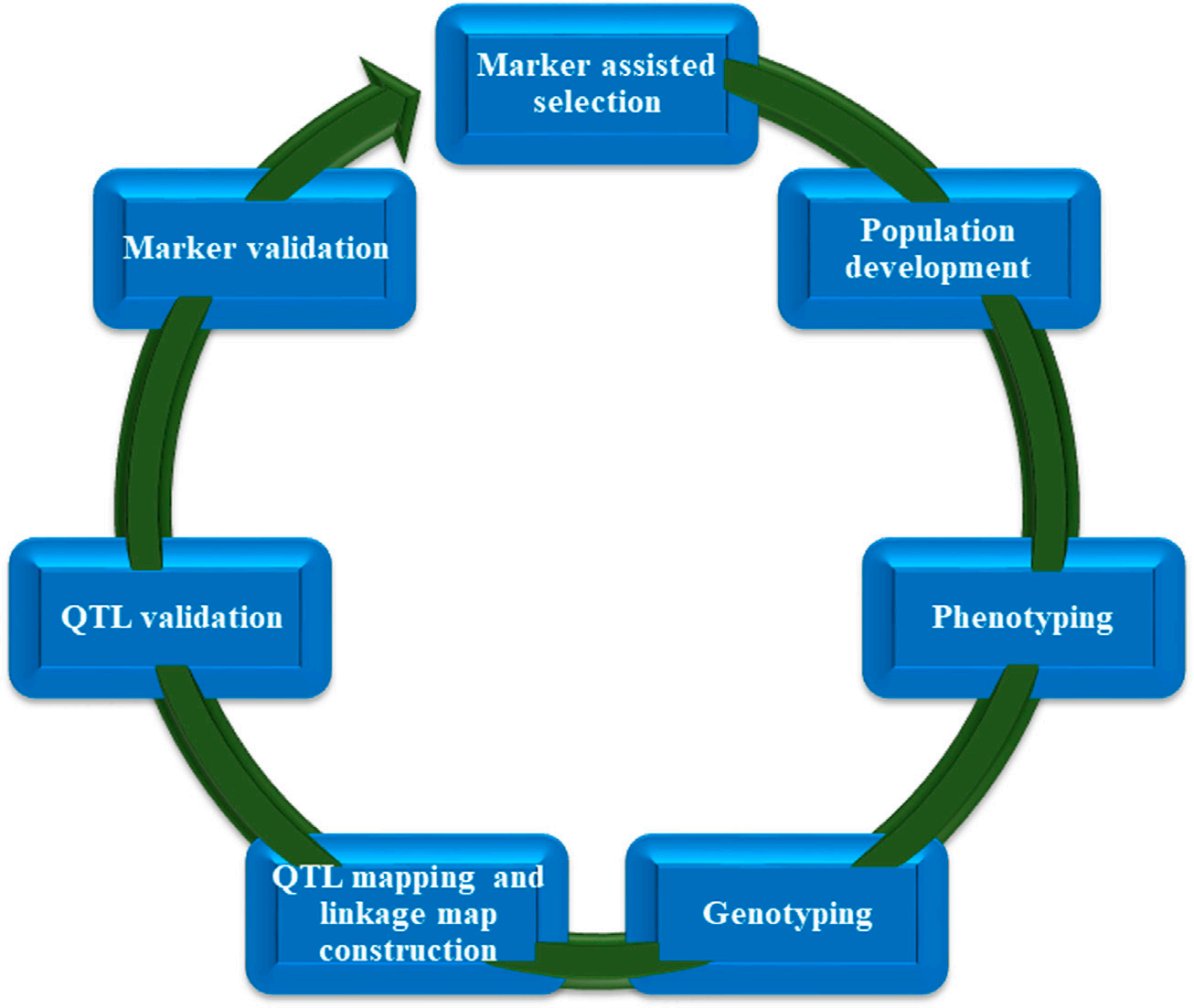
Important stages of marker-assisted selection (MAS).

The selection of parents for crossover can be made using genomics-assisted breeding, as can the validation of the genetic purity of F1 offspring, the mapping of features for introgression, and the molecular profile of breeding populations for the purpose of selecting enhanced types. Molecular markers have seen widespread usage in the study of cultivated and raced sorghum in order to ascertain the level of genetic variation present in the crop (Deu et al., 1994; Oliviera et al., 1996; Menkir et al., 1997). Sorghum hybrids and cultivars have been developed during the past few decades using traditional breeding techniques. They have increased grain yield and greater flexibility. In order to long-term advance of sorghum to fulfill the needs of the future, continual progress in the gene engineering of important adaptive and productive characteristics is required. The yield and efficiency of sorghum have just reached a plateau, and the efforts that have been put into breeding are becoming less fruitful. Potential productivity can be reduced by factors like vulnerability to water shortage, pests and diseases, and weeds, particularly striga. Traditional breeding to improve these complicated features has not yielded adequate results, owing to the intricate genetic control of these traits as well as significant environmental influence. However, if the selection is based on DNA markers, rapid genetic increases can be accomplished (Bernardo, 2014). DNA markers are thus becoming increasingly important for the genetic improvement of sorghum. Multiple marker systems have been developed and applied to tag and map significant influence genes and quantitative characteristics (QTL) of economic importance, i.e., grain yield and its components, insect pest and disease resistance, Striga, salinity, drought, nutritional quality and cold, among others. Some important steps involved in marker assisted selection (MAS) are described in Figure 3.

### Biotic stress in sorghum

The sorghum crop is threatened by numerous biotic factors, including infectious pathogens/diseases, parasitic weeds and insect pests. Annually, biotic diseases, alone or in combination with other factors, cause a substantial economic loss in sorghum (Mengistu et al., 2019). Presently, one hundred and fifty insect species have been recorded in sorghum crops, among which the most destructive are aphid species (*Rhopalosiphum maidis* and *Melanaphis sacchari*), *Atherigona soccata* (Shoot-fly), and *Stenodiplosis sorghicola* (Sorghum midge) (Kumari et al., 2021). Weed infestation in sorghum significantly reduces its yield. The wild *Striga* is the common weed restrain sorghum yield in all continents, especially in Africa. In heavily infested soils, Striga spp. can reduce crop yields by up to 100% (Kavuluko et al., 2021). The use of modern genetically modified methods to recognize and transfer candidate genes to improve productivity and resistance to biotic stress has the potential to boost overall sorghum performance (Hariprasanna and Rakshit, 2016). It is important to identify and use new genes for host plant resistance in order to develop biotic stress-resistant cultivars.

### Marker-assisted breeding in sorghum for biotic stresses

Sorghum, like other cereal crops, is susceptible to biotic stresses such as bacterial, fungal, nematode, and virus infections. The most common diseases are fungal infections caused by smut, charcoal rot, ergot, downy mildew, blast, and rust, which have a greater impact on sorghum plant health and productivity (Fakrudin et al., 2021). Other common fungal diseases that affect sorghum fodder and grain production include blast (*Pyricularia oryzae*) and rust (*Puccinia purpurea*). Rust has caused significant yield reductions of 50%, 30%, and 13.1% in India, the Philippines, and Australia, respectively (White et al., 2012). To develop resistance in sorghum against the phyllosphere fungal diseases, Sharma et al. (2012) tested 242 sorghum accessions in both *in vitro* and *in vivo* conditions. The findings of this study reveal that six out of 242 accessions (IS 33023 (Tanzania), IS 23521 (Ethiopia), IS 473(US), IS 23684 (Mozambique), IS 24503, and IS 26737 (South Africa) showed resistance against sorghum foliar fungal diseases.

Four bacterial diseases, i.e., leaf spot (*Pseudomonas syringae*), leaf streak (*Xanthomonas campestris* pv. *Holcicola*), leaf strips (*Burkholderia andropogonis*) and red stripe (*Herbaspirillum rubrisubalbicans*) considerably affect the sorghum plant (Kutama et al., 2010; Tuleski et al., 2020). The rain, wind, and humid wet conditions are favorable for the dispersal of bacterial infection from a single plant to a pandemic. Plant parasitic nematodes (PPN) are also posing a negative impact on sorghum crop production (Traore et al., 2012). Sorghum crops are affected by a wide range of PPN, including *Xiphinema americanum* (Dagger), *Meloidogyne* spp. (Root knot), *Paratrichodorus minor* (Stubby root), *Tylenchorhynchus* spp. (Stunt), *Longidorus africanus* (Needle) and *Belonolaimus longicaudatus* (Sting). The infected sorghum plant becomes stunted at the initial stage of root infection and delays blooming, consequently destroying the overall harvesting yield (Little et al., 2018).

Like other infectious diseases, viral diseases also have a deleterious effect on sorghum plant health and yield. Several viruses affect sorghum globally, causing several symptoms (Oksal et al., 2021). There are 23 viruses that have been identified worldwide as pathogenic to sorghum plants, with nine (09) of them being found in Asian countries. These sorghum viruses are divided into three sections, Potyvirus: Johnson grass mosaic virus, sugarcane mosaic virus, sorghum mosaic virus, and maize dwarf mosaic virus; Rhabdovirus: maize mosaic virus and Tenuivirus: Maize Stripe Virus which negatively affect the sorghum crop in different regions of the world (Srinivas et al., 2010). Sorghum shows the symptoms of delayed flowering, mosaic, chlorotic streaks, mottling, reddening of leaves, yellowing, dwarfing, necrotic spots, and sterility in response to viral diseases (Li et al., 2013; Achon et al., 2017). Unfortunately, up to now, none of the sorghum lines are showing genetic resistance against the reported viruses. To improve the resistance of sorghum against viral diseases, there is a dire need to modify the genome of the host plant and detect the QTLs by using GWAS and other molecular markers (Mengistu et al., 2021; Nida et al., 2021). To combat plant disease, an integration of disease management strategies, such as chemical methods and cultural methods, can be beneficial. Sorghum’s tolerance to biotic stresses has been improved with the help of genomic techniques, including large scale genotyping and high-throughput sequencing. The use of marker-assisted breeding and transgenics will support more effectively addressing the problem of diseases (Madhusudhana, 2019). A number of resistant genes in sorghum plants against all the above-mentioned diseases have been identified previously by using different molecular markers (Table 5).

**TABLE 5:** QTL identification for resistance to plant diseases.

Fungi	Disease	Linked marker	Chr. position	QTL/Gene name	References
	Grain mold	Xtxa10057	SBI-09	PR genes (QTL gene not shown)	Klein et al. (2001)
		Xtxp12	SBI-04		
		Xtxa1047	SBI-10		
		Xtxa10062	SBI-07		
		Xtxp95	SBI-06		
		S4_62316425	Chr.1	TAN1 gene	Nida et al. (2021)
		Chr2_5600094	SBI-02	Rxo1 and Hin1	Upadhyaya et al. (2013)
		Chr8_8997626	SBI-08		
	Anthracnose	rs1887698	Chr.9	Sobic.009G126300	Mengistu et al. (2021)
		rs1938969	Chr.6	-	
		rs100028710	Chr.4	-	
		rs2681689	Chr.9	Sobic.009G008800	
		rs5196058	Chr.9		
		rs100052771	Chr.1		
		rs1884746	Chr.10	Sobic.010G012200	Ahn et al. (2019)
		SNPs	Chr.8	Calmodulin isoform 4	Tesso et al., 2012
		AFLP	SBI- 05	Cg1	
	Charcoal rot	SB2437	Chr.4	SbSCP3 SbA1E, SbBGL5 and SbCDPK1	Mahmoud et al. (2018)
		SbAGB03	Chr.9	SbCCR SbPG, TuMAPK, SbJAR1 and SbHIR1	
		Xtxp287	Chr.9	SbEPRL, SbLEC14B, StRGA2, SbTIR1, SbRPP13, and SbERS2	
	Rust	SNP markers	Chr.6	Rp1-dp3 and rph1-3	Wang et al. (2014)
	Head smut	SSRs	Chr.1 and Chr.5	NB-LRR	Ahn et al. (2019)
	Ergot	TxP274	SBI-02	PR gene	Parh et al. (2008)
		Sb6-84	SBI-06		
	Target leaf spot	1020 SNP markers	Chr.5	ds1	Kimball et al. (2019)
Bacteria	Red stripe	SNPs markers	Chr.7	SORBIDRAFT_07g020210	Tuleski et al. (2020)
			Chr.3	SORBIDRAFT_03g007910	
Nematode	Southern root-knot nematode	SNPs markers	Chr. 1	NBS-encoding genes	Wang et al. (2014)
		SNPs markers	Chr.3	QTL-Sb.RKN.5.1.	Harris-Shultz- et al. (2019)
			Chr.5	Sobic.005G055900	
		SSRs markers	Chr.3	QTL-Sb.RKN.3.1	Harris-Shultz et al. (2015)
Virus	Sorghum mosaic virus (SrMV)	SSRs markers	RNAi	anti-SrMV	Guo et al. (2015)

### Insect pest

The quick resistance and mutation in the genomes of various insect species against synthetic insecticides have become a major challenge to gaining maximum yield. The sorghum plant is a host to 150 insect species that are suppressing the plant’s efficacy and playing a role in yield loss (Guo et al., 2011). The lepidopterous Chilo partellus (stem borer), dipterans Stenodiplosis sorghicola (midges), Atherigona soccata (shoot flies), and hemipteran *Schizaphis graminum* (Greenbug) are the most damaging pests of sorghum (Okosun et al., 2021). Tao et al. (2003) explained two genetic mechanisms (antixenosis and antibiosis) of resistance in the cross sorghum line ICSV745 × 90,562 against the midge. Identification and characterization of genetic loci through marker-assisted breeding and map-based cloning involved in *S. graminum* resistance. The 36 QTLs involved in resistance to *S. graminum* have been registered in many studies (Table 6) (Wu and Huang, 2008; Punnuri et al., 2013). Shoot fly (Atherigona soccata) is also considered a major damaging pest of sorghum. The larvae of A. soccata cut the new emerging points of the shoot and caused symptoms like a dead heart. Polymorphisms in sorghum’s resistance to shot flies have been found, and these polymorphisms have been exploited to locate genetic loci that affect sorghum’s resistance to shoot flies. A large number of molecular traits and resistance loci are identified in various sorghum genotypes and cultivars using the molecular SSRs, SNPs, and GWAS approaches, including SFCR 151, IS 1057, PS 30715–1, IS 1071, IS 1082, IS 4663, IS 1096, IS 2312, IS 2146, IS 18369, IS 2205, IS 18551, and IS 2394 against *A. soccata*. Up to this point, only a select few resistant germplasms have been utilized in *A. soccata* resistance breeding initiatives (Chamarthi et al., 2011; Kumari et al., 2021).

**TABLE 6:** Details on insect pest resistance loci discovered in sorghum.

Green bug	Population, size and Biotype	Linked marker	QTL gene	PVE (%)	Chr.	References
	F2, 371 & I	Starssbnm93-Starssbem286	Qstsgrsbi09ii	39.8	SBI-09	Punnuri et al. (2013)
		Starssbem296-Starssbnm93	Qstsgrsbi09i	64–82	SBI-09	
		Starssbnm102-Starssbem298	Qstsgrsbi09iii	34	SBI-09	
	RILs, 88, & I,K	Xtxp12	VIS-GBK1	9 to 19	SBI-04	Nagaraj et al. (2005)
		Xtxp335	IS-GBK5		SBI-05	
		Xtxp43	SPA-I2		SBI-05	
		Xtxp335	SPA-K2		SBI-05	
		Xcup20	VIS-GBK2		SBI-04	
		Sb1-10	VIS-GBI8		SBI-04	
	F2, 277 & I	Xtxp230-Xtxp67	QSsgr-09–02	54–80.3	SBI-09	Wu and Huang (2008)
		Xtxp358-Xtxp289	QSsgr-09–01		SBI-09	
	F2:3, 233 & E	Xtxp289-Xtxp358	-	58–84.8	SBI-09	Wu et al. (2007)
	F3, 370	pSB347	Ssg1	4–78	SBI-09	Katsar et al. (2002)
	F3, 203 & C,E	pSB262-pSB089	Ssg3		SBI-05	
	F3, 489 & C	CSU030	Ssg2		SBI-08	
	F3, 195 & C,I,E, K	pSB443	Ssg4		SBI-03	
	RIL, 93 & K, I	B18-885	-	5.6–38	SBI-01	Agrama et al. (2002)
		OPB12-795			SBI-05	
Shoot-fly	RIL & 210	Xtxp99-Xcup57	QTdu.dsr-7 QTdl.dsr-10.1 QTdu.dsr-10 QTdl.dsr-10.2	4.2	SBI-07 SBI-10 SBI-10 SBI-10	Aruna et al. (2011)
		Xtxp320-Xgap1		24.1		
		Xcup16-Xtxp320		44		
		Trit-Xtxp320		12.6		
	RIL & 254	Xtxp248–Xtxp316	-	6.2	SBI-01	Apotikar et al. (2011)
	168 & RIL	Xnhsbm1013-Xnhsbm1044	QTdu.dsr-10.1 QTdl.dsr-1.2 QTdl.dsr-6 QGs.dsr-5	15.7	SBI-10 SBI-01 SBI-06 SBI-05	Satish et al. (2009)
		Xtxp302-Dsenhsbm80		5.3		
		Xtxp274-Xtxp317		5.2		
		Xtxp30-Xtxp6		14		
	RIL & 168	XnhsbmSFC34	Cysteine protease Mir1	22.1	SBI-10	Satish et al. (2012)
	F1 and F2	Ch8_20519857-Ch8_20217087	qRE8.1	44.35	SBI-02 SBI-06	Pandian et al. (2020)
		Ch4_48400958-Ch4_46023941	qVI4.0	21.23		

### Parasitic weeds

Parasitic weeds are a major cause of sorghum yield reduction around the world (Bellis et al., 2020). The losses due to this biotic factor have been recorded at up to 60% (Dille et al., 2020). Different weeds like crabgrass (Digitaria sanguinalis), common lamb squarters (*Chenopodium album*), palmer amaranth (Amaranthus palmeri), johnsongrass (Sorghum halepense), common waterhemp (Amaranthus tuberculatus), shattercane (Sorghum bicolor ssp. verticilliflorum), kochia (Bassia scoparia), and Striga (S. hermonthica) have been recorded in various regions of the world (Peerzada et al., 2017). Striga sp. is the most dangerous weed for the sorghum crop. The losses due to Striga weeds alone in Africa were recorded at 30%–100% (Mrema et al., 2016). Teka (2014) conducted a research trial for the screening of different sorghum germplasms for Striga resistance and concluded that the germplasms Framida, N13, Seguetana CZ, SRN39, PSL5061, ICSVs, P9401, SRN39, 555, PSL5061, CMDT45, P9403, P9403, P9401, and Soumalemba are resistant to Striga. There is a need to find and develop genomic-based resistance in sorghum against Striga. QTL mapping is a vital source for finding resistance genes in sorghum against these parasitic weeds. Haussmann et al. (2004) used SSR markers combined with MAS and revealed 6 resistance QTL genes in sorghum against the Striga from Chromosome A, Chromosome J1, Chromosome B, Chromosome I, and Chromosome J2. Resistant QTL and MAS-based studies have the efficacy to aid in the development of resistant cultivars. A key prerequisite for breeding programs is knowledge of Striga resistance in host plants and the genetic basis for this resistance. According to hypothesis, Striga resistance in sorghum is due to a combination of factors such as incompatibility, hypersensitive reactions, low haustoria initiation activity, and low germination stimulant activity. Cowpea was the first plant for which the Striga-resistant gene was discovered. This discovery was made by Li et al. (2009). To date, several QTLs have been identified in sorghum for Striga resistance (Table 7).

**TABLE 7:** QTLs identified in sorghum for Striga resistance.

QTL/gene	Chromosomes	Source of resistance	References
97 QTL	SB1, 2, 3, 4,6, 7, 9, 10	19 accessions	Kavuluko et al. (2021)
*Lgs*	SBI-05	SRN39	Satish et al. (2012)
*HR2*	SBI-05	*S. arundinaceum*	Grenier et al. (2007)
*Lhf*	SBI-09	PQ434	Grenier et al. (2007)
9 QTLs	SBI-01, 07, 06, 4,03, 02	N13	Haussmann et al. (2004)
HR1	SB1-02	*S. arundinaceum*	Mohamed et al. (2003)

### Abiotic stress tolerance

Tolerance to abiotic stress is intricate and often controlled by a large number of quantitative trait loci (Mwamahonje et al., 2021; Osman et al., 2022). The generation of superior lines with enhanced resilience to abiotic stress using molecular breeding procedures has a limited track record of success (Tonapi et al., 2020). Due to its significance, several QTL mapping work for stay green in sorghum have been documented (Table 8) (Kiranmayee et al., 2015; Aquib and Nafis, 2022). So far, 7 stay green trait source have been exploited to identify QTLs for this phenotype. These included QL41 (Tao et al., 2000), SC283 (Sabadin et al., 2012), B35 (Harris et al., 2007), SDS 1948-3 (Habyarimana et al., 2010), E36-1 (Haussmann et al., 2002), 296B (Srinivas et al., 2009a), and SC56 (Kebede et al., 2001). The most prevalent of these sources is B35 (Rosenow et al., 1983). Stay green was found to be quantitatively inherited in all studies, however the QTLs diverse across years and environments. Moreover, 6 main stg QTLs (i.e., stg C, stg 3A and stg 3B, stg 1 and stg 2, and stg4) on SBI-01, SBI-02, SBI-03, and SBI-05, respectively, have been uncovered via various examinations (Tao et al., 2000; Xu et al., 2000; Haussmann et al., 2002; Sanchez et al., 2002; Harris et al., 2007). The molecular studies recognized four major stay green QTLs designated as Stg1, Stg2, Stg3, and Stg4, as well as several minor QTLs (Sabadin et al., 2012). Stg1 and Stg2 were mapped to sorghum chromosome 3, explaining approximately 20%–30% of the phenotypic variance (Sanchez et al., 2002; Harris et al., 2007). The Stg3 gene is found on chromosome 2, and the Stg4 gene is found on chromosome 5. Stg3 and Stg4 are responsible for 16% and 10% of the morphological traits, separately (Sanchez et al., 2002; Harris et al., 2007).

**TABLE 8:** QTLs identified for abiotic stress tolerance in sorghum.

Stress type		Name of the parents	No. of QTLs	Traits	References
Drought		76T1-23× Baji, 76T1-23× Birmash and Meco-1 × Birmash	105	DST and OATs	Techale et al. (2022)
		BC2F1 × BC2F3	18	DT, STG	Mwamahonje et al. (2021)
		Not available	3	DT	Abou-Elwafa and Shehzad (2018)
		TX7078×B35	15	HS, YS and YPS	Tuinstra et al. (1996)
		TX7078×B35	16	SWS, YS, SG and GY	Tuinstra et al. (1997)
		B35× Tx430	9	SM and SG	Crasta et al. (1999)
		B35× Tx7000	24	SG and CC	Xu et al. (2000)
		B35× Tx7000	7	SG and CC	Subudhi et al. (2000)
		QL39× QL41	10	SG	Tao et al. (2000)
		SC56× Tx700	35	SG, PFDT, PH, FT and LT	Kebede et al. (2001)
		IS9830 × E36–1 andN13 × E36-1	10	SG	Haussmann et al. (2002)
		BTx642 × Tx7000	4	SG	Hayes et al. (2016)
Salinity	2.0% NaCl	Shihong137 × L-Tian	24	RL, SH, SFW, TFW, RFW, SDW, RDW TDW	Wang et al. (2014)
	0.6% NaCl	Shihong137 × L-Tian	31	SD, PH, StFW, TB, JW and Brix	Wang X. et al. (2020)
Low nitrogen	Low N (0 kg ha^−1^)	CK60 × China17	20	DAS, PH, CC, HMC, SMC, BY, GY, TW, and GSR	Gelli et al. (2016)
	High N (100 kg/ha)		18		
	Low N (0 kg/ha)	CK60 × China17	10	CC, PH, DAS, SMC, HMC, BY, GY, TW and GSR	Gelli et al. (2017)
	High N (100 kg/ha)		11		
Low phosphorus	Low P (0 kg/ha)	BR007 × SC283	17	GY, RD and SAFR	Bernardino et al. (2019)
Cold	13 C	Shan Qui Red × SRN39	2	Seed germination	Knoll et al. (2008)
	12 C	RTx430 × PI610727	14	PSG, FE, and SV	Burow et al. (2011)

HS, height stability; YS, yield stability; YPS, yield per plant; SWS, seed weight stability; SG, stay green; GY, grain yield; SM, seed maturity; CC, chlorophyll content; TDW, total dry weight; PFDT, post-fowering drought tolerance; FT, flowering time; LT, lodging tolerance; RL, root length; SH , shoot height; TFW, total fresh weight; RFW, root fresh weight; RDW, root dry weight; SD, stem diameter, StFW, stem fresh weight; TB, total biomass; JW, juice weight; DAS, days to anthesis; HMC, head moisture contents; PH, plant height; SMC, stover moisture content; BY, biomass yield; GY, grain yield; TW, test weight; GSR, grain to stover ratio; SAFR, surface area of fine roots; SV, seedling vigor; SDW, shoot dry weight; PSG, percentage of seed germination; FE, field emergence; RD, root diameter; SFW, shoot fresh weight.

Sorghum production in tropical and subtropical climates faces a substantial constraint due to Al toxicity (Gao et al., 2021), which is the primary factor impacting crop production on two-thirds of the acid soil affected area (Guimaraes and de Magalhaes, 2021; Rahman and Upadhyaya, 2021). When the Al-sensitive BR007 is crossed with the Al-tolerant SC283, only one gene (i.e., SbMATE/AltSB) controls Al tolerance in sorghum. This gene is located on chromosome 3 (Magalhaes et al., 2004). Concerning temperature, generally sorghum cannot grow well when the temperature is less than 15°C. Early planting may be possible, particularly in the United States, if sorghum cultivars can be induced with early-season cold tolerance. This will allow the range in which sorghum may be grown to be enlarged to include more northern latitudes (Yu and Tuinstra, 2001). Bekele et al. (2013) found a highly interacting epistatic QTL hotspot that had a significant effect on longer chilling survival. They discovered numerous genes that confer cell division and growth maintenance in response to early freezing stress within QTL hotspot regions, which might be potential candidates to breed cold resistance. According to the findings of Balota et al. (2010), a higher respiration rate in sorghum is associated with better germination despite the presence of cold stress. Washburn et al. (2013) associated a rhizome development trait with sorghum’s ability of overwintering. Overwintering and rhizomatousness phenomena are governed by 7 QTLs as reported by Paterson et al. (1995) and Washburn et al. (2013) which were recognized in a mapping population of BTx623/S. propinquum. Anami et al. (2015) revealed that sorghum’s potential to overwinter and develop rhizomes are advantageous for biofuel production.

Salinity is also a growing issue in sorghum cultivated areas in several parts of the world (Mansour et al., 2021; Punia et al., 2021; Ukwatta et al., 2021). A sum of thirty eight QTLs affecting salt tolerance have been recognized from an RIL population including 181 lines originated from a cross between L-Tian and Shihong 137 (Wang et al., 2014). Six of these QTLs have been identified as major QTLs, accounting for more than 10% of phenotypic variation. Salt tolerance mechanism differs between germination through seedling stages. More research is required before applying the recognized QTLs in MAS. Due to the unpredictability of the timing, duration, and intensity of drought occurrence in the natural environment, as well as the difficulties of constructing screening environments, traditional breeding techniques have made direct selection for drought resistance components slow and challenging. Consequently, the use of molecular markers in QTL analysis based on meticulously managed repeatable testing has the ability to overcome difficulties associated with variable and unpredictable onset of moisture stress or the confounding influence of other related stresses such as heat (Ejeta et al., 2000). Four QTLs conferring pre-flowering drought tolerance in sorghum from RILs derived from the cross (SC 56 Tx7000), however, theses QTLs are inconsistent across environments. Numerous stay green QTLs related to post-flowering drought tolerance have been mapped and molecular markers associated to these QTLs are available (Sanchez et al., 2002; Kassahun et al., 2010). Post-flowering drought stress is also referred to as “stay green” since these plants retain chlorophyll in their leaves and can sustain photosynthesis for longer in comparison to “senescent” genotypes under drought stress.

In comparison to pre-flowering drought stress, more QTLs related to several morphological traits have been recognized under post-flowering drought stress. By using 170 RFLP and RAPD markers, a set of 98 RILs derived from a cross between B35 x TX7078 were utilized to identify 5 QTLs for yield stability, 3 for seed weight stability, 2 for grain yield and 6 for stay green under post flowering drought stress (Tuinstra et al., 1997). By using 125 RILs, derived from Tx700 and SC56 and 170 RFLP markers under drought, QTLs controlling plant height (6 QTLs), flowering time (5 QTLs), pre-flowering drought tolerance (7 QTLs), lodging tolerance (3 QTLs), stay green (14 QTLs) were recognized in sorghum (Kebede et al., 2001). For proper plant growth, development and higher yield, fourteen nutrients are necessary and each of these nutrients has a particular role in developmental processes of plants (Narayan et al., 2022). QTLs and transporters for other nutrients-related traits such as Mg, Mo, Cl, Mn, Zn, Co, Ni, and Fe have not yet been recognized. There are less studies in sorghum related to nutrient usage efficiency in comparison to rice, wheat, and maize. More studies should be carried out to uncover QTLs and transporters for all of these nutrients in order to increase nutrient usage efficiency in sorghum.

### Marker-assisted breeding for agronomic traits

As sequencing and phenotyping tools continue to improve rapidly, a number of key genetic loci and genes regulating sorghum agronomical traits have been found, mostly by genome-wide association studies, quantitative trait locus mapping, and mutant analyses (Kaur et al., 2021; Chakrabarty et al., 2022; Niu et al., 2022). The major agronomical traits that affect sorghum yield are tiller numbers, grains per panicle, and grains weight (Somegowda et al., 2022). Around 340 QTLs related to grain yield are recognized in sorghum (Mace et al., 2019). In a panel of 354 accessions with 265,487 SNPs, nine hotspots related to seed weight were identified (Zhang et al., 2015). Boyles et al. (2016) detected 53, 19, and 36 significant SNPs related to grain number per main panicle, 1000 grain weight, and grain yield per main panicle, respectively. A large-scale GWAS research was conducted on a panel of 837 accessions of sorghum and a BC-NAM population of 1421 populations, being able to isolate grain size 81 QTLs (Tao et al., 2020). In order to develop early flowering sorghum crops, the Sorghum Crop Improvement Program (SCIP) has made use of prominent sorghum accessions such as BTx406 and SM100, both of which hold recessive alleles of Sbprr37 and Sbghd7. In addition, it has been found that genes such as SbMED12, LD and SbSUC9 are connected to the process of maturation (Upadhyaya et al., 2013). Two significant QTLs on LG1 are associated with protein digestibility; one QTL (connected with Xtxp11) negatively affects protein digestibility, whereas the second QTL (linked with Xtxp88) 20 cm distant positively affects protein digestibility (Winn et al., 2009). Four main loci controlling sorghum plant height have been identified (i.e., Dw1, Dw2, Dw3, and Dw4) of which Dw2 and Dw3 show pleiotropic effects. Significant progress has been made in the identification of genetic loci affecting renewable energy-related traits in sorghum. A key characteristic of sweet sorghum is its juicy stem (sugar-rich). In association analysis of traits related to sugar including sugar volume, midrib color, and 42,926 SNPs related to sugar yield, Burks and colleagues (2015) found a significant relationship at a distance of 51.8 Mb (on chromosome 6), which corresponds to a region that contains a dry midrib locus. Panicle length, width, weight, the number of primary branches per panicle, the number of seeds per panicle, and the panicle harvest index are regarded as the most significant components of panicle directly influencing the total grain yield (Hmon et al., 2013). Sorghum’s inflorescence architecture has been studied less than that of other Poaceae species (Witt Hmon et al., 2014; Nagaraja Reddy et al., 2013). A major QTL, QPle-sbi06-2, between markers GlumeT-Xxp145 on LG 6 that accounts for more than fifty percent of the variation in panicle length was identified (Srinivas et al., 2009a). This QTL is also co-located with the major height QTL (Dw2) and the major maturity gene (Ma1). Major QTL (>10%) for panicle length have been identified, with 12 being meta-QTL with phenotypic variation ranging from 5.91%–50.4% discovered in various genetic backgrounds (Nagaraja Reddy et al., 2013; Zou et al., 2012; Witt Hmon et al., 2014). Another trait associated with grain yield is panicle width, for which seven major QTL have been identified (Hart et al., 2001). With 14%–20% phenotypic variation, five major QTLs pertaining to primary and secondary seed branches have been recognized in diverse genetic backgrounds (Brown et al., 2006; Srinivas et al., 2009b). Four major loci influencing sorghum plant height have been identified (Dw1, Dw2, Dw3, and Dw4), with Dw2 and Dw3 exhibiting pleiotropic effects. Following linkage and linkage disequilibrium mapping, numerous quantitative trait loci (QTL) for plant height in sorghum were discovered (Higgins et al., 2014; Hilley et al., 2016). QTL with significant effects on plant height have been identified in a variety of genetic backgrounds and have been linked to qualitative loci, Dw1 on SBI-09, Dw2 on SBI-06, and Dw3 on SBI-07 (Morris et al., 2013a; Nagaraja Reddy et al., 2013; Higgins et al., 2014; Zhao et al., 2016). The map position of Dw2 on SBI-06 on the consensus map strictly linked with DArT markers, sPB-7169 and sPB-1395, and Dw3 between a simple sequence repeat marker, mcbCIR300 and a DArT marker, SSCIR57 on SBI-07 was registered (Mace and Jordan, 2011). The map position of Dw4 has not yet been reported. Morris et al. (2013b) suggested that the Dw2 phenotype is caused by a loss of function in a sorghum histone deacetylase gene (Sobic.006G067600) that controls plant height in other crops such as rice, maize, and Arabidopsis. Some major QTLs identified for agronomic traits are given in Table 9.

**TABLE 9:** QTLs identified for agronomic traits in sorghum.

Traits	Quantitative trait locus (gene)	Encoding protein	Phenotype	Gene ID	References
	Unknown	γ-Kafirin protein	SP	Sobic.002G211700	De Freitas et al. (1994)
	Unknown	δ-Kafirin protein	SP	Sb10g013050	Izquierdo and Godwin (2005)
	Unknown	β-Kafirin protein	SP	Sobic.009G001600	Chamba et al. (2005)
	Sh1	YABBY transcription factor	SS	Sobic.001G152901	Lin et al. (2012)
	Unknown	α-Kafirin protein	SP	Sobic.005G193000	Wu et al. (2013)
	SbWRKY	WRKY transcription factor	SS	Sobic.001G148000	Tang et al. (2013)
	qGW1	Expressed protein	GW	Sobic.001G038900	Han et al. (2015)
	Unknown	Similar to H0801D08.10 protein	GS	Sb06g033060	Zhang et al. (2015)
	Unknown	Similar to putative fibre protein Fb34	GS	Sb10g018720	Zhang et al. (2015)
Grain quality	KS3	Ent-kaurene synthase	SN	Sb06 g028210	Zhao et al. (2016)
	MSD1/SbTCP16	TCP (Teosinte branched/Cycloidea/PCF) transcription factor	GNP	Sobic.007G135700	Jiao et al. (2016)
	MSD3	ω-3 Fatty acid desaturase	GNP	Sobic.001G407600	Dampanaboina et al. (2019)
	y1	MYB domain protein	PC	Sobic.001G398100	Boddu et al. (2005)
	Wx	Granule-bound ADP-glucose-glucosyl transferase	ET	Sobic.010G022600	Sattler et al. (2009)
	Tannin1/B2	WD40 protein	Testa	Sobic.004G280800	Morris et al. (2013b)
	DGAT1	Diacylglyceroal O-acyltransferase 1	CF	Sobic.010G170000	Boyles et al. (2017)
	AMY3	Alpha-amylase debranching enzyme	FPC	Sb02g023790	Rhodes et al. (2017b)
Flowering and height	Ma3	Phytochrome B	Maturity	Sobic.001G394400	Childs et al. (1997)
	Dw3	Auxin efflux transporter	PH	Sobic.007G163800	Multani et al. (2003)
	Ma1/SbPRR37	Pseudoresponse regulator protein 37	Maturity	Sb06g014570	Murphy et al. (2011)
	LD	Flowering-time protein	Maturity	Sb03g045030	Upadhyaya et al. (2013)
	SbMED12	Flowering regulator	Maturity	Sb01g050280	Upadhyaya et al. (2013)
	SbSUC9	Sugar transporter	Maturity	Sb06g000520	Upadhyaya et al. (2013)
	Ma6	CCT-domain protein	Maturity	Sb06g000570	Murphy et al. (2014)
	Dw1/SbHT9.1	Membrane protein	PH	Sobic.009G229800	Hilley et al. (2016)
	Dw2	Protein kinase	PH	Sobic.006G067700	Hilley et al. (2017)
	RAP2-7	Ethylene responsive transcription factor	PH	Sobic.009G024600	Girma et al. (2019)
Brown midrib and stem texture	bmr6	Cinnamyl alcohol dehydrogenase	BM	Sobic.004G071000	Saballos et al. (2009)
	bmr12	Caffeic O-methyltranferase	BM	Sobic.007G047300	Sattler et al. (2012)
	bmr2	4-Coumarate: coenzyme A ligase	BM	Sb04g005210	Saballos et al. (2012)
	SbSWEET4-3	Sugar transporters	SA	Sobic.004G136600	Mizuno et al. (2016)
	SbVIN1	Vacuolar invertase	SA	Sobic.004G004800	McKinley et al. (2016)
	SbTST2	Tonoplast sugar transporters	SA	Sb04g008150	Bihmidine et al. (2016)
	SbTST1	Tonoplast sugar transporters	SA	Sb01g030430	Bihmidine et al. (2016)
	SbSWEET8-1	Sugar transporters	SA	Sobic.008G094000	Mizuno et al. (2016)
	Dry	NAC transcription factor	DSM	Sobic.006G147400	Zhang et al. (2018)
Tillering	tb1	Transcription factor	Tillering	Sobic.001G121600	Kebrom et al. (2010)

SP, seed protein; SS, seed shattering; GW, grain weight; GS, grain size; SN, seed number; GNP, grain number per panicle; PC, pericarp color; ET, endosperm texture; CF, crude fat; FPC, fat and protein content; PH, plant height; BM, brown midrib; SA, sugar accumulation; DSM, dry stem and midrib.

### Marker-assisted breeding for nutritional and quality traits

Sorghum has lower protein digestibility than other cereal grains, which is thought to be due to disulfide cross-linking in the b- and g-kafirins. Two major QTL on LG 1 are related with protein digestibility: one QTL (linked with Xtxp11) has an unfavorable effect on digestibility, while another QTL (linked with Xtxp88) 20 cm away has a favorable effect o digestibility (Winn et al., 2009). Association mapping for grain quality in a diverse sorghum collection identified SNPs in several genes (Sukumaran et al., 2016). An SNP on the candidate gene SSIIa located on LG 10 was associated with kernel hardness and explained 8% of the variation in the trait. SNPs associated with kernel hardness (SB00214.1 and SB00214.2) explained 10% of variation in the trait and they were located in the locus pSB1700 on LG 3. SNPs SB00156.1 and SB00054.1 associated with calcium content were located in LG 3 at 50 and 59 cm, respectively. SB00156.1 was located in a locus, pSB0289, which was predicted to produce serine/threonine protein kinase. An SNP on the starch synthase IIb (SSIIb) gene on LG 4 was found to be significantly associated with starch content and explained 10% of the variation in the trait. Significant progress has been made in the identification of genetic loci affecting renewable energy-related traits in sorghum. A key characteristic of sweet sorghum is its juicy stem (sugar-rich). In association analysis of traits related to sugar including sugar volume, midrib color, and 42,926 SNPs related to sugar yield, Burks et al. (2015) found a significant relationship at a distance of 51.8 Mb (on chromosome 6), which corresponds to a region that contains a dry midrib locus.

Ritter et al. (2008) conducted an analysis of QTL for stem sugar-related and other agronomic traits using a population derived from sweet sorghum (“R9188”) and grain sorghum (“R9403463-2-1”). QTL were recognized for all sugar traits and were largely co-located to five locations (SBI-01, SBI-03, SBI-05, SBI-06, and SBI-10). For sucrose content, three major QTL were consistently detected on SBI-06, which were also co-mapped with sugar content and Brix. A major QTL was also detected on SBI-05 related to SSR marker, mSSCIR12, where a major QTL for sugar content also co-located. Two major QTLs for fructose and sucrose content were co-located on SBI-06 near Xtxp547 marker explaining 18%–24%, respectively. Calviño et al. (2008) compared genes in grain (BT 623) and sweet (Rio) sorghum to identify genes involved in sugar accumulation and lignocellulose synthesis. In sweet sorghum, “Rio,” 132 transcripts were downregulated and 63 were upregulated. A saposin-like type B gene displayed the highest level of differential expression among the upregulated transcripts in “Rio.” Numerous transcripts related to carbohydrate metabolism were upregulated, such as hexokinase 8; sorbitol dehydrogenase; carbohydrate phosphorylase; and NADP-malic enzyme. Transcripts that were downregulated included sucrose synthase 2 and fructokinase 2, a- and b-galactosidases, and several others related to cell wall activities like cellulose synthase 1, 7, 9 and cellulose synthase catalytic subunit 12 involved in cellulose synthesis. Furthermore, a series of genes encoding proteins in lignin synthesis like cinnamyl alcohol dehydrogenase, cinnamoyl-CoA reductase, caffeoyl-CoA O-methyltransferase, 4-coumarate:coenzyme A ligase were also downregulated. Bihmidine et al. (2016) investigated the expression of two additional classes of sucrose transport proteins, Tonoplast Sugar Transporters and SWEETs, for sucrose accumulation in sweet sorghum stems and validated the differential expression of these two genes in grain and sweet sorghum stalks.

### Functional markers

Advancements in molecular biology, such as genomics, genome editing, and high-throughput sequencing, make cultivar development faster and more precise (Salgotra et al., 2014). Recognizing genes and functional markers (FMs) that are strongly associated with plant phenotypic variation is a difficult task. FMs have an advantage over other plant breeding markers due to their close genomic association with phenotypes. Consequently, FMs may facilitate the direct selection of genes associated with phenotypic traits, thereby enhancing the selection efficiencies required to develop varieties. Using marker-assisted selection (MAS) techniques, FMs are utilized in precision breeding for agronomic and quality traits as well as breeding for abiotic and biotic stress resistance. FMs are DNA markers that have been derived from functionally defined sequence motifs (Anderson and Lubberstedt, 2003). Therefore, SNPs as FMs are superior to RDMs and genic molecular markers (GMMs) in plant breeding. Although GMMs may exist within a gene of interest, they may not be functionally linked to the phenotypic trait of interest, which could result in false selection in MAS.

### FMs for the improvement of agronomic traits, quality traits, and stress resistance

Advancement in sequencing techniques allow the identification of SNPs and indels related to several economically essential traits; FM development is thus enabled (Kage et al., 2016). Indels may cause phenotypic variation as a result of extensive genomic effects, which are accompanied by the possibility of elimination from natural selection. Therefore, SNP-derived FMs are superior to indel-derived markers due to the widespread distribution of FMs throughout the genome (Li et al., 2012). Various FMs for agronomic, quality, and biotic and abiotic stress resistances have been developed (Table 10) and pyramided in various crops using MAS, MABB, MARS, and GS techniques (Kage et al., 2016). Quality characteristics are essential for a variety of reasons, including satisfying consumer preferences. FMs have been successfully utilized in MAS to enhance the nutritional value of crops. In sorghum, an InDel-based FM for the SbBADH2 gene responsible for fragrance has been developed. This FM has been utilized to identify sorghum genotypes with high fragrance, which can then be utilized in sorghum breeding (Zanan et al., 2016). An FM has been developed for the SbMATE gene, which confers aluminum stress tolerance in sorghum (Too et al., 2018). The SbMATE FM assists in screening sorghum germplasm for aluminum stress tolerance, which can then be used in breeding. Li et al. (2016) reported that in sorghum, FM tightly linked to the seed dormancy QTL may be used in marker-assisted selection for seed dormancy.

**TABLE 10:** Functional molecular markers (FMMs) developed for various traits in sorghum.

Traits	Gene/Locus	Alleles/polymorphic sites/marker name and nature of polymorphism	Trait to which FMMs developed	References
Disease traits	Cs1A and Cs2A	Functional nucleotide based markers	Anthracnose resistance	Biruma (2013)
	Sorghum ESTs	Sorghum ESTs derived SSR markers	Disease resistance	Savadi et al. (2012)
Quality traits	Sorghum unigenes	Unigene derived SSR markers	Regulatory and functional proteins	Nagaraja Reddy et al. (2012)
	GBSS	1 bp, 5 bp deletion based CAPS marker	Waxy phenotype	Lu et al. (2013)
	SbBADH2	1, 444 bp deletion based marker	Fragrance	Yundaeng et al. (2013)
	SAI-1	SAI-1a (132 bp), SAI-1b (136 bp),SAI-1c (141 bp); deletion based	Soluble acid invertase	Liu et al. (2014)

### Transcriptomics

Transcriptomics is uncovering the differential expression of genes in a biological system. Transcriptome analysis innovations such as massively parallel signature sequencing (MPSS), sequencing-based approaches (RNAseq) and microarrays, have made it possible to comprehend the transcriptomic alterations that occur under various developmental or environmental stress conditions. Analysis of these transcriptomic alterations has yielded a comprehensive understanding of the cellular and molecular responses involved in plant development in response to stress tolerance (Johnson et al., 2014). RNA-Seq facilitates the identification and quantification of transcripts via a high-throughput sequencing assay. In addition to quantifying gene expression over a wider dynamic range, this method is extremely useful for identifying alternative splicing events (Wang et al., 2019). Even though it has been the most popular method for transcript profiling in numerous crop species, it fails detecting multiple full-length transcripts that are reconstructed from short-read sequences (Steijger et al., 2013; Wang et al., 2016). Because of that limitation, RNA-Seq is inadequate for examining gene regulation, the protein-coding capacity of the genome, and ultimately phenotypic diversity. Advanced techniques such as Oxford Nanopore and PacBio Single Molecule Sequencing are better suited to identify comprehensively full-length transcripts because they directly generate full-length cDNA sequences (Wang et al., 2016). Abdel-Ghany et al. (2016) sequenced the BTx623 sorghum transcriptome, using Pacific Biosciences singlemolecule real-time sequencing, resulting in the identification of transcriptome-wide full length isoforms with >11,000 novel splice isoforms and alternative splicing and alternative polyadenylation (APA) ∼11,000 expressed genes and >2,100 novel genes, enhancing sorghum genes annotation. Sorghum, as one of the few climate-resilient crops, has an inherent ability to adapt to climate change, particularly during severe abiotic stress conditions (i.e., drought, elevated temperature, salinity … ) (Carpita and McCann, 2008). This makes sorghum a model of choice for understanding the molecular mechanisms involved in stress adaptation (Mace et al., 2013). Advances in NGS technologies, as well as the availability of complete genome sequences of several sorghum genotypes, provide excellent opportunities for studying molecular mechanisms at the transcriptome level (Mace et al., 2013). Determining the transcriptional response of sorghum to both drought and heat stresses individually and in combination by Johnson et al. (2014) using microarrays consisting of 28,585 gene probes exposed differential expression of genes to the tune of ∼4% and 18% following drought and heat stresses, respectively, while ∼20% genes revealed differential expression in response to combined stress.

This study demonstrated evidence of sorghum’s specific response to individual stresses as well as crosstalk to combined heat and drought stresses. Sorghum plants with the stay-green trait can retain green leaf area even during maturity under drought conditions and yield more than their senescent counterparts. A comparison of gene expression between stay-green (B35) and senescent (R16) cultivars for the purpose of elucidating the molecular and physiological basis of drought tolerance revealed that the differentially expressed transcripts were associated with the response to osmotic stress. Specifically, the expression of delta1-pyrroline-5-carboxylate synthase 2 (P5CS2) was higher in the stay-green line as compared to senescent line, and this high expression showed correlation with higher proline levels (Johnson et al., 2015). A comparative transcriptome analysis between two cultivars (623B and Henong 16) under the imposition of salt treatment (0.8% NaCl) for 0, 48, and 72 h conducted by Cui et al. (2018) reported a total of 5647 differentially expressed genes (DEGs). Functional annotation of these DEGs showed that majority of them are involved in regulation of transcription, signal transduction, and secondary metabolism, mainly genes encoding kinases and transcription factors. Zhang et al. (2019) identified 510, 559, and 3687 DEGs in leaves and 3368, 5093, and 4635 DEGs in roots that responded to mild drought, severe drought, and re-watering treatments, respectively, in an RNA-Seq-based transcriptomic profiling of sorghum leaves and roots under drought. Among them, 190 common DEGs in leaves and 1644 common DEGs in roots were responsive to mild drought, severe drought, and re-watering environment. According to gene ontology (GO) enrichment analysis, these genes are linked with water deprivation, abscisic acid stimulation, and reactive oxygen species.

Furthermore, genomic regions enriched with drought-responsive genes encoding heat shock protein (HSPs), expansin, and aquaporin could be used as potential targets for genetic improvement of drought tolerance in sorghum. A study on sorghum’s tolerance to progressive water deficit and re-watering using a South African landrace (LR6) and cDNA microarrays containing 35,899 transcript probes revealed 902 transcripts that were differentially expressed in response to the aforementioned treatments (Devnarain et al., 2019). Among the 26 genes recognized to be involved in response to abiotic stimulus, the recognition of β-alanine betaine in sorghum leaf extracts and substantial increase in its relative abundance during severe stress highlighted the involvement of β-alanine betaine biosynthesis in imparting drought tolerance in sorghum establishment and seedling growth. Chopra et al. (2015) used RNA-Seq with control and cold stress treatments to profile the transcriptomes of cold-sensitive (BTx623) and cold-tolerant (HongkeZi) sorghum lines in order to comprehend the molecular mechanism underlying cold tolerance. The study discovered transcription factors like dehydration-responsive element-binding factors, C-repeat binding factors, and ethylene-responsive transcription factors that were significantly upregulated in cold-tolerant lines during cold stress. Furthermore, under cold stress, differential regulation of genes such as plant cytochromes, glutathione s-transferases, and heat shock proteins was observed between cold-tolerant and cold-sensitive lines. In an other study by Marla et al. (2017) comprising of RNA sequencing of seedlings of a chilling-tolerant Chinese accession along with a chilling-sensitive US reference line and mass spectrometry of four chilling-tolerant Chinese accessions along with two US reference lines indicated chilling-induced upregulation of C-repeat binding factor (CBF) (cold-response regulator) and genes involved in detoxification of reactive oxygen, biosynthesis of jasmonic acid, and phospholipase Da1 (PLDa1) (lipid remodeling gene) in the chilling-tolerant Chinese accession. Moreover, the results showed the involvement of CBF-mediated transcriptional regulation, galactolipid and phospholipid remodeling, and jasmonic acid responsible for chilling adaptation in Chinese sorghums. Precise annotation of transcriptional unit and its expression pattern is vital for transcriptome analysis, and a collection of full-length cDNA (FL-cDNA) facilitates this. A normalized FL-cDNA library was constructed in sorghum from eight different growth stages of aerial tissues; 37,607 clones were isolated and sequenced to obtain 38,981 expressed sequence tags (ESTs). A total of 272 novel genes, 323 antisense transcripts, and 1672 candidate isoforms were annotated, and the expression of 70.6% of these novel genes were confirmed by spikelet-, seed-, and stem-specific RNA-Seq analysis. A transcriptome database (MOROKOSHI) was created with this data along with 23 sorghum RNA-Seq data available in the public domain and was displayed on a genome browser (Makita et al., 2014). SorghumFDB, a platform for functional annotations of genome and multidimensional network analyses, was developed by Tian et al. (2016), which includes annotations of whole genome assemblies, miRNA sequences and their targets, common gene families, gene networks using transcriptome data, as well as annotation elements for multiple gene function. This is useful for studying each gene’s expression profile in order to identify a group of genes with the most similar expression. Visualization tools like Cytoscape, Gbrowse, and open-flash-chart as well as sequence analysis tools like GSEA, BLAST, motif significance analysis, and pattern set were integrated in the database for the determination of functional prediction. These databases will aid in understanding the functional relationships between genes, gene co-expression, and improving the accuracy of functional genomics analyses, resulting in a better understanding of gene regulatory networks involved in sorghum genetic improvement.

### Application of GWAS in sorghum

The accomplishment of molecular plant breeding is heavily restricted to a small number of characteristics governed by major impact genes as a result of the inherent drawbacks of low genome insurance of molecular markers, obstacles in locating an enormous list of biological variation, and the inescapable linkage drag associated with the selection of suitable regions of chromosomes. Molecular plant breeding is currently only feasible for a select few of these characteristics (Gupta et al., 2010). Additionally, in order to speed breeding procedures, advances in theoretical framework as well as technological advancements are required. Development of next-generation sequencing, genome editing, genome selection, molecular modules, non-invasive high throughput phenomics, and GWAS has revolutionized plant breeding’s scope by facilitating the development of numerous new tools enabling the more efficient exploitation of previously restored natural or artificially created variations (Chen B. R. et al., 2019; Godwin et al., 2019; Xu et al., 2020).

The GWAS is an effective approach for determining the inheritance pattern of complex agronomical traits by applying a single nucleotide polymorphism (SNP) biomarker that has been effectively employed in the investigation of the genetic basis of complex agronomical traits. GWAS has been used to identify significant genomic regions and genes that control important complex traits in sorghum (Table 11). Morris et al. (2013b) created the most widely used panel and genotyped it using genotyping-by-sequencing (GBS), yielding a total of 265,487 SNPs. This panel was then used to explore the genetic basis of yield and its related traits like plant height, inflorescence morphology, grain quality traits, stalk rot and anthracnose (Colletotrichum sublineolum) disease resistance (Cuevas et al., 2018; Cuevas et al., 2019; Hao et al., 2021). Increasing crop adaptation to environment is essential for guaranteeing sustainable food supply since environment has a considerable impact on agricultural performance. In regard to the previously described highly complicated agronomical traits, extensive research on G x E interaction in sorghum has been initiated. Since its domestication, sorghum has spread extensively across varied agroecological zones, and it is easy for studying G x E interaction. Lasky et al. (2015) described the allelic associations with bio-climatic and soil contours in a collection of 1943 sorghum accessions which were investigated using 104,627 SNPs. Results disclosed that factors related to environment could illustrate a significant portion of SNP alteration and genic SNPs were boosted for environmental (Al toxicity and drought) associations. They proposed that SNP integration knowledge could be used to assess and adapt to traits in a given environment. Another study by Olatoye et al. (2018) regarding genome-wide association mapping of precipitation parameters discovered a minor but substantial involvement of clinical modification, which is influencing nucleotide alteration in Nigerian sorghum germplasms and revealed that the genes inherent morphology with photoperiod fluctuation play important roles in adaptation against drought.

**TABLE 11:** Genome Wide Association Studies (GWAS) study in sorghum.

Abiotic stress	Marker types	Chr. No	Traits	*p*-values	References
Drought	SNPs	6,7	Flowering time plant height, grain weight, forage biomass, drought tolerance, water use	-	Maina et al. (2022)
	SNPS	6	1000-grain weight, flowering time plant height, grain weight and drought Tolerance	-	Faye et al. (2022)
	SNPs		Pre and post flowering drought stress, vegetative biomass, fresh total plant biomass, plant height and leaf area index	-	Spindel et al. (2018)
	SNPs	1	Leaf senescence, drought tolerance and plant height	-	Wang X et al. (2020)
	SNPs	6	Plant height	-	Morris et al. (2013b)
	SNPs		plant height	-	Ongom (2016)
Heat stress	SNP	2,5,6	fresh shoot weight	-	Chopra et al. (2017)
	SNP	4	Chlorophyl Content	-	Chopra et al. (2017)
		3,6	Shoot length	-	Chopra et al. (2017)
	SNP	2	Leaf firing	*p* = 1.15 × 10^−7^	Chen et al. (2017)
	SNP	9	leaf blotching (LB)	*p* = 7.28 × 10^−8^	Chen et al. (2017)
Inflorescence traits	SNP	3	Absence or Presence of awns	*p* ≤ 3.05000E-11	Girma et al. (2019)
	SNP	2,5,6	Compactness of the panicle and its form	*p* ≤ 4.38953E-10	Girma et al. (2019)
	SNP	6	Panicle exsertion	*p* ≤ 4.78977E-08	Girma et al. (2019)
	SNP	1,2,3,4,5,7,8,10	Panicle exsertion	*p* ≤ 4.86 × 10^−5^	Zhao et al. (2016)
	SNP	1,3,4	Pericarp color	*p* ≤ 3.33216E-14	Girma et al. (2019)
	SNP	6	Glume cover	*p* ≤ 1.38672E-06	Girma et al. (2019)
	SNP	1	Chlorophyll (SPAD)	1.5E-05	Tefera (2019)
Cold (seedling stage)	SNP		Amino acid biosynthesis	-	Chopra et al. (2017)
	SNP	2,6	Anthocyanin content and root length	-	Chopra et al. (2017)
	SNP	3,6	Shoot length, shoot weight and root weigh	-	Chopra et al. (2017)
	SNP	3, 4, 6, 7, and 8	Gas exchange and chlorophyll fluorescence traits	-	Ortiz et al. (2017)
Agronomic traits	SNP	1, 3, 5, 6, 7, 8, 9 and10	Plant height and maturity	-	Habyarimana et al. (2022)
	SNP	8.9	Heading date	-	Habyarimana et al. (2022)
	SNP	3,8,9	Dry/fresh biomass	-	Habyarimana et al. (2022)
	SSR	2,6,9	Plant height	-	Wang et al. (2012)
	SNP	10	Days to flowering	*p* ≤ 1.01 × 10^−6^	
	SNP	1,3,5,9	Days to flowering, Panicle width, Panicle length and grain yield	-	Enyew et al. (2022)
	SNP	1,3,6,8,9	Panicle length, panicle width	-	Tefera (2019)
	SNP	2	Grain weight	-	Boyles et al. (2016)
	SNP	5.6	Hundred seed weight	2.5E-10	Tefera (2019)
				3.3E-07	
	SNP	6,7,9	Pre-flag height	-	Morris et al. (2013b)
	SNP	2,5,7,8,9	Plant height	*p* ≤ 3.33852E-08	Girma et al. (2019)
	SNP	3,8,9	Plant height	5.11 × 10^−6^	Luo et al. (2020)
				3.77 × 10^−6^	
				1.78 × 10^−7^	
	SNP	6,9	Plant height	*p* ≤ 2.67 × 10^−7^	Zhao et al. (2016)
	SNP	5,7,8	Plant height	-	Girma et al. (2019)
	SNP	2, 6, 8, and 9	Tiller number	2.68 × 10^−6^	Luo et al. (2020)
				3.61 × 10^−7^	
				1.07 × 10^−7^	
				5.51 × 10^−6^	
	SNP	1,3,4,8,9,10	Tiller number	*p* ≤ 1.77 × 10^−6^	Zhao et al. (2016)
	SNP	3,5	Tillers per plant	2.1E-08	Tefera (2019)
				5.8E-10	
	SNP	1, 2, 8, and 10	Panicle length	1.26 × 10^−8^	Luo et al. (2020)
				6.11 × 10^−6^	
				3.83 × 10^−6^	
				3.63 × 10^−7^	
	SNP	3,5,10	Panicle length	*p* ≤ 1.69 × 10^−6^	Zhao et al. (2016)
	SNP	3	Grain yield	-	Leiser et al. (2014)
	SNP	1	Grain mold resistance	-	Nida et al. (2019)
Disease		1,8,10	Grain mold resistance	−log (*p*-value) = 6.18,	Cuevas et al. (2019)
				−log (*p*-value) = 6.88,	
				−log (*p*-value) = 5.84	
	SNP	1	Anthracnose resistance	0.000000104	Ahn et al. (2022)
	SNP	5	Anthracnose resistance	1.39 × 10–7	Cuevas et al. (2018)
	SNP	3,6,8	Smut resistance score	*p* ≤ 4.27118E-08	Girma et al. (2019)
	SNP	9	Stalk rot resistance	3.01E-04	Adeyanju et al. (2015)
	SNP	4	Starch	3.66E-07	Kimani et al. (2020)
Quality traits	SNP	1,5,6,9	Starch contents	-	Chen K. et al. (2019)
	SNP	4	Flavonoid Pigmentation Traits	-	Morris et al. (2013b)
	SNP	4	Tannin content	*p* = 1.62E-08	Kimani et al. (2020)
	SNP	4	Tannin content	-	Rhodes et al. (2017a)
	SNP	5,6,8	Brix	8.12 × 10^−7^	Luo et al. (2020)
				3.56 × 10^−7^	
				6.02 × 10^−6^	

Zm, zea mays; Os, oryza sativa; ubi 1, ubiquitin1, nptII; Ta, *Triticum aestivum*; Act1, actin 1; CaMV, cauliflower mosaic virus; bar, bialaphos resistance; neomycin phosphotransferase II.

Faye et al. (2019) used 213,916 SNPs in 421 Senegalese sorghum germplasm accessions to analyze genomic variation and domains altered by environmental issues. This was done by analyzing the genomic differences between the accessions. Flora transcription factors along with stay green aided in climate tolerance in the Sudanian and Sahelian zones. Wang J. et al. (2020) collected a total of 1901 georeferenced sorghum accessions and the correlation study of seed bulk variation with precipitation gradient proved that seed bulk variation was responsive to precipitation gradient. Detailed genomic understanding of adaptive features will aid in predicting how well sorghum will perform in various environmental situations. Adaptive and agronomic features are complicated in nature and influenced by a number of environmental and the genetic factors. The collected data typically shows that the majority of genetic loci can only account for a modest portion of phenotypic variation. We should be aware that descriptive statistics and models, which are largely used in GWAS, may result in erroneous negative or positive results. GWAS results can all be impacted by SNP frequency, phenotypic precision, population structure, and population size. However, GBS, which is prone to producing significant amounts of missing data, accounts for the majority of the genetic variants in sorghum during the studies by (Annicchiarico et al., 2015). Consequently, a bigger size of the population and a greater SNP mass are required. Appropriate statistical techniques must also be established to deal with greater marker frequencies and larger sample sizes. Conventional GWAS frequently screens out uncommon variants with MAF ≥ 0.05, which may represent morphological variance, in favor of the common variants with MAF < 0.05 that are the primary focus of the study. So, it is important to come up with a unique statistical and association research method for these unusual differences in order to fill the gaps in information.

### Genetic transformation in sorghum

**FIGURE 4:**
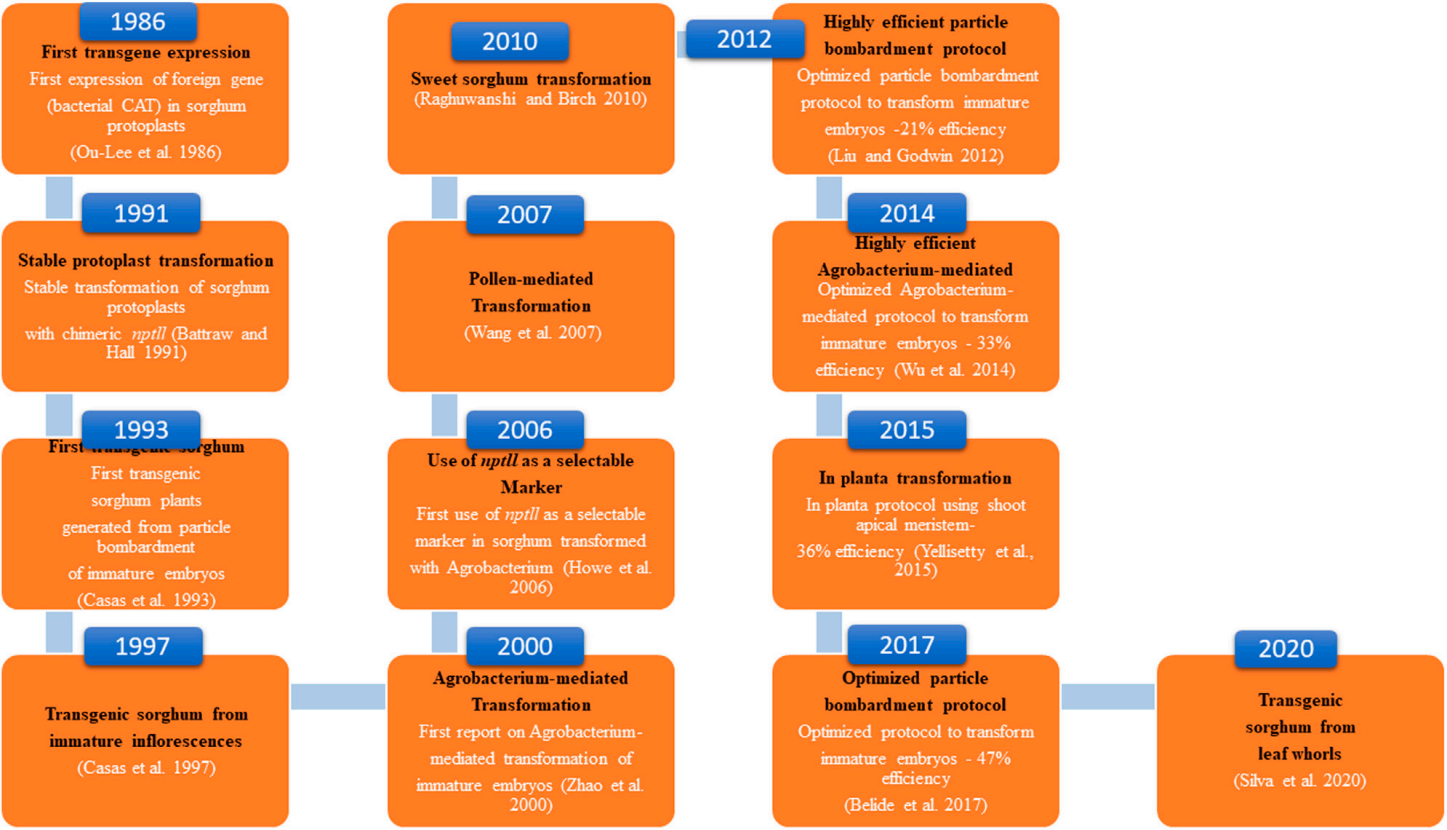
Sorghum transformation progress timeline.

The inability to transform sorghum is a key obstacle to its broad usage as a bioenergy source in the emerging bio-economy and in a research planning system. Sorghum transformation is difficult from a technological standpoint, expensive, and time-consuming, and it can only be achieved for few genotypes. Because of its genotype-dependent responses, short-term plant regeneration capacities, production of phenolic chemicals and acclimation challenges, sorghum showed resistant to transformation during tissue culturing (Maheswari et al., 2006; Altpeter et al., 2016). Since the introduction of the concept of transgenic sorghum, a great deal of progress has been accomplished (Casas et al., 1993) (Figure 4). Casas et al. (1993) achieved a 0.3 percent transformation efficiency by inducing callus development for particle bombardment in immature embryos from the genotype P898012. Since then, by using the genotype Tx430, the process has been enhanced to the point where it now achieves up to 46.6% efficiency (Belide et al., 2017). When the transgene is introduced by inoculation with *Agrobacterium tumefaciens*, the efficiency of transformation has enhanced from 9.7% to 33%. (Zhao et al., 2000; Wu et al., 2014). Selection of genotype is an essential part of tissue culture and, as a result, transformation success. Because of its exposure to elevated regeneration frequency and callus induction, the grain sorghum inbred line known as Tx430 has seen a significant uptick in usage over the course of the past decade (Wu et al., 2014; Belide et al., 2017). However, Tx430 was associated with 7 bioenergy parental sorghum lines using the protocols from Wu et al. (2014) and Liu and Godwin, (2012). The line Tx430 showed a high callus multiplication but a moderate phenolic release, whereas the lines Rio and PI329311 had the highest regeneration rates as reported by Flinn et al. (2020).

## Novel biotechnological approaches for sorghum breeding

### Genome editing

Emerging genome editing technologies provide a unique opportunity to boost agricultural output and quality through the targeted change of genes that influence traits like stress tolerance and nutrient uptake (Kamthan et al., 2016; Anwar and Kim, 2020). The pioneer work on sorghum genome editing was initiated by Jiang et al., 2013. The DsRED2 specific coding region was targeted by designing a 20 bp specific guide RNA (sgRNA). The integration of guide RNA was followed under the control of the U6 promoter from rice. For selectable and visual markers, the GFP-NptII gene was integrated with the expression vector under the control of the CaMV 35s promoter. Two weeks post-transformation, GFP expression was detected in groups of cells, showing the presence of stably transformed cells. Five of the 18 cell groups which were GFP-positive also expressed DsRED2. This approaches the theoretical maximum frequency of one-third for reading frame rescue of DsRED2 by NHEJ, suggesting that the gene editing in transformed cells was highly efficient. However, although Jiang et al. (2013) were successful in establishing gene editing using CRISPR/Cas9, the target gene was co-introduced, and was therefore not endogenous to sorghum. The edited cells were not regenerated into plants and sequencing evidence of the edits was not provided. The first report of successful endogenous gene editing in sorghum was reported by Che et al. (2018) who improved the transformation method by using a tripartite vector system to boost efficiency. This technique was used to mutagenize the specific histone H3 of sorghum at centromere locus. One successful example of editing whole gene families in sorghum was reported by Li et al. (2018). This study was performed to enhance sorghum grain quality. Both the Cas9 as well as the sgRNA were deployed inside of a binary vector (pBUN421), which was then driven by promoters for maize ubiquitin and rice U3, respectively. For the purpose of effective co-targeting of the complete gene family, a singular sgRNA was developed with the intention of focusing on the extremely conserved N-terminal ER signal. This was the first study in which multiple targets were edited successfully with one sgRNA in sorghum. As seen in Table 12, a high editing efficiency of 92.4% was reported. In this context, it is important to note that the study targeted 20 genes with one sgRNA, only one of which needed to be edited for the event to be considered an edited event, which contributed to the elevated editing efficiency. The studies discussed thus far all employed a single sgRNA cassette for gene targeting. However, multiplex gene editing has also been demonstrated using two guide RNAs to Table 1 simultaneously target two distinct endogenous genes (Char et al., 2020a). A stable knockout of the cinnamyl alcohol dehydrogenase (CAD) gene in the variant Tx430 was created utilizing the biolistic delivery method reported by Liu et al. (2019) employing a particle inflow gun (PIG). A 25% editing efficiency was reported, but no information about the lines was given because the publication just described the technique. The delivery of genome editing tools to sorghum via Agrobacterium has been described in two studies (Sander, 2019; Char et al., 2020b).

**TABLE 12:** List of genome editing studies in sorghum.

Promoter sgRNA/Cas	No of gRNA	Delivery method	Target gene	SM	Edit efficiency (%)	Phenotype	Reference
OsU6/OsAct1	1	Agrobacterium	mDsRED2	nptII	NR	DsRED2 expression	Jiang et al. (2013)
ZmU6/ZmUbi1	1	Agrobacterium	Sb-CENH3	nptII	37–40	NR. Biallelic frameshift mutations potentially lethal	Che et al. (2018)
TaU3/ZmUbi	1	Agrobacterium	k1C gene family	nptII	92.4	Partial opacity in T1 seeds, reduced α-kafirin, improved grain protein digestibility and lysine content	Li et al. (2018)
OsU6/ZmUbi1	2	Agrobacterium	SbFTSbGA2ox5	bar	33.3, 83.3	Delayed flowering. No phenotype, biallelic mutations potentially lethal	Char et al. (2020b)
OsU6/CaMv35S	2	Agrobacterium	SbLG1	nptII	33.3	Altered leaf inclination angle, ligule and auricle size. Distinct phenotypes for WT, monoallelic and biallelic mutants	Brant et al. (2021)

### Genomic selection

Genomic selection (GS) is one effective breeding tool in the selection of complex quantitative traits, like yield. That tool has been effectively applied in plant breeding (Meuwissen et al., 2016) and is substantially attaining recognition among plant breeders, specifically for those traits that are challenging to evaluate (Crossa et al., 2017; Xu et al., 2020). Sorghum has proven to be one of the best crop for using genomic selection because of the availability of the reference genome sequence and genomic resources. Nonetheless, genomic selection for sorghum improvement is less prevalent than for wheat and maize. For the first time, genomic selection in sorghum targeting plant height demonstrated that predicted plant height based on UAV sensing had a strong correlation with measured plant height. Several recent articles regarding genomic selection for sorghum improvement (de Oliveira et al., 2018; Hunt et al., 2018; Habyarimana and Lopez-Cruz, 2019; Velazco et al., 2019) have revealed the potential of this approach for achieving genetic gain in sorghum breeding programs. In sorghum, GS primarily focused on model training using a range of training populations like natural population, mixed populations, and testcross hybrids (Yu et al., 2016; Habyarimana et al., 2020). In addition, more research incorporating phenotypic and genotypic data from natural or breeding populations should be conducted to reveal the genetic mechanisms governing several critical agronomic traits. Sorghum genetic diversity that has been conserved in the form of germplasm allows for the identification of new alleles and genes responsible for conferring desirable traits. It is necessary to preform phenotypic and genotypic characterization on the entire germplasm of sorghum in order to do full justice to its collection as well as conservation efforts. It will result in fully characterized raw material that can be used for genetic improvement. This would also aid with parent selection, identifying genes and markers for all prominent traits, and achieving the required degree of trait expression through genomic selection. This tool has the ability to enable sorghum cultivators, all involved sectors, and the industry to reach more profitable outcomes. Breeders of forage sorghum can use a comprehensive DNA fingerprint to evaluate the genetic potential of untested individuals, allowing them to make genomic predictions for use in selection. It allows to shorten the breeding cycle, improve selection precision, and increase the rate of genetic gain in forage species. A panel of 976 sorghum accessions were genotyped utilizing 0.72 million SNPs obtained using genotyping-by-sequencing for a study involving GS for biomass traits. A prediction model was developed to predict the biomass-related attributes of the remaining untested germplasm using the collection of 300 best representative accessions that were phenotyped for these variables (Yu et al., 2014). It is obvious that GS is a potential tool for crop improvement in the near future and can modernize the traditional method of plant breeding. The availability of high throughput phenomics platforms will help this even more.

### Future outlooks

Improving agricultural production is critical to ensure food security and to meet the needs of the continuously increasing world population. Particularly, meeting the food demand of developing countries in Africa and Asia is quite challenging. Among other crops, sorghum also holds good position in fulfilling the feed and food requirements of poor people in various countries in Africa and Asia. Abiotic stresses severely affects sorghum production across the world. Marker Assisted Breeding (MAB) is a promising tool, among others, in the framework of modern plant breeding. MAB has established itself as a powerful tool for genetic manipulation of crops for crop improvement by means of agronomic traits, qualitative and quantitative traits, and biotic and abiotic stress resistance. MAB provides numerous notable advantages over traditional phenotype-based selection techniques. It can be efficiently used in germplasm characterization, QTL mapping, gene pyramiding, genetic diversity, and evolutionary and phylogenetic studies. To date, the practical use of MAB in plant breeding has been confined to simple traits with monogenic or oligogenic inheritance. Through MAB, genetic improvement of traits with complicated inheritance remains a challenge. Factors like poor resolution of QTLs on the genetic map, a lack of robust markers, imprecise estimates of QTL locations and effects, non-validation of marker-trait associations, genetic background, epistasis, a lack of cost-effective marker genotyping systems, G x E interactions, a knowledge gap in plant breeders, a lack of wet lab facilities, and other factors can all contribute to the low visible impact of marker assisted breeding. Aside from drought stress, very few QTLs have been discovered in sorghum under abiotic stress conditions. There are numerous desirable agronomic attributes best represented by sorghum, however, the genes and QTLs for these traits have yet to be uncovered. Numerous root QTLs have been explored in maize and rice but no efforts in this regard under abiotic stresses have been made in sorghum. More research is needed to uncover QTLs for root, agro-morphological, and yield-related attributes that could help to increase sorghum growth and yield. Genome editing technologies like CRISPR, which have lately gained popularity, may be useful in creating mutants for improved sorghum performance under abiotic stresses. In general, the traits of sorghum for abiotic stress tolerance have still to be investigated however, few functional genomics and molecular breeding studies have been started but a thorough understanding of these studies is necessary to enhance the development and yield of sorghum under stresses. Sorghum is considered to be a model crop among cereals for comparative genetics to reveal different physiological mechanisms underlying drought and heat tolerance due to its small genome size, wide diversity and germplasm resources, whole-genome sequence availability, array of marker systems and high-density linkage maps. Several studies have revealed QTLs for numerous attributes, and the integration of linkage maps has produced saturated consensus maps. Several of these QTL have been validated in various genetic contexts and are therefore appropriate for marker-assisted breeding in sorghum. For attaining higher genetic gains, it is necessary for plant breeders to integrate MAB into conventional breeding tools. The association of MAB with next-generation sequencing, cisgenetics, epigenetics, and CRISPR technology for genome editing can lead to the establishment of a new platform of low-cost, high-throughput crop improvement in the coming years. In the future, innovations in cost-effective and more precise molecular breeding are likely to be seen for the precise and quick development of new potent plant varieties by effective incorporation of novel traits and improvement in economically important plants.
